# Adaptive Remodeling of the Bacterial Proteome by Specific Ribosomal Modification Regulates *Pseudomonas* Infection and Niche Colonisation

**DOI:** 10.1371/journal.pgen.1005837

**Published:** 2016-02-04

**Authors:** Richard H. Little, Lucia Grenga, Gerhard Saalbach, Alexandra M. Howat, Sebastian Pfeilmeier, Eleftheria Trampari, Jacob G. Malone

**Affiliations:** 1 Department of Molecular Microbiology, John Innes Centre, Norwich Research Park, Norwich, United Kingdom; 2 School of Biological Sciences, University of East Anglia, Norwich Research Park, Norwich, United Kingdom; 3 The Sainsbury Laboratory, Norwich Research Park, Norwich, United Kingdom; University of California, Santa Barbara, UNITED STATES

## Abstract

Post-transcriptional control of protein abundance is a highly important, underexplored regulatory process by which organisms respond to their environments. Here we describe an important and previously unidentified regulatory pathway involving the ribosomal modification protein RimK, its regulator proteins RimA and RimB, and the widespread bacterial second messenger cyclic-di-GMP (cdG). Disruption of *rimK* affects motility and surface attachment in pathogenic and commensal *Pseudomonas* species, with *rimK* deletion significantly compromising rhizosphere colonisation by the commensal soil bacterium *P*. *fluorescens*, and plant infection by the pathogens *P*. *syringae* and *P*. *aeruginosa*. RimK functions as an ATP-dependent glutamyl ligase, adding glutamate residues to the C-terminus of ribosomal protein RpsF and inducing specific effects on both ribosome protein complement and function. Deletion of *rimK* in *P*. *fluorescens* leads to markedly reduced levels of multiple ribosomal proteins, and also of the key translational regulator Hfq. In turn, reduced Hfq levels induce specific downstream proteomic changes, with significant increases in multiple ABC transporters, stress response proteins and non-ribosomal peptide synthetases seen for both Δ*rimK* and Δ*hfq* mutants. The activity of RimK is itself controlled by interactions with RimA, RimB and cdG. We propose that control of RimK activity represents a novel regulatory mechanism that dynamically influences interactions between bacteria and their hosts; translating environmental pressures into dynamic ribosomal changes, and consequently to an adaptive remodeling of the bacterial proteome.

## Author Summary

Post-transcriptional control of protein abundance is a significant and underexplored regulatory process by which organisms respond to environmental change. We have discovered an important new mechanism for this control in bacteria, based on the covalent modification of a small ribosomal protein by the widespread enzyme RimK. Here we show that the activity of RimK has specific effects on the levels of ribosomal proteins in the cell, which in turn affects the abundance of the important translational regulator Hfq. RimK is itself controlled by binding to the small regulatory proteins RimA and RimB and the widespread signalling molecule cyclic-di-GMP. Deletion of *rimK* compromises motility, virulence and plant colonisation/infection in several different *Pseudomonas* species. We propose that changes in intracellular RimK activity enable *Pseudomonas* to respond to environmental pressures by changing the nature of their ribosomes, leading in turn to an adaptive phenotypic response to their surroundings. This promotes motility and virulence during the initial stages of plant contact, and phenotypes including attachment, metabolite transport and stress control during long-term environmental adaptation.

## Introduction

Post-transcriptional mechanisms for the regulation of protein abundance are critical for the control of diverse cellular processes including metabolism and nutritional stress responses [1,2], virulence and antibiotic production [3] and quorum sensing [4]. In addition to well-studied pathways for mRNA translational control by proteins such as RsmA and Hfq [4–6], riboswitches [1], and direct ribosomal interference [2], a further potential regulatory mechanism is the specific alteration of ribosome function by posttranslational modification of its associated proteins. Numerous ribosomal proteins undergo posttranslational modifications including methylation, acetylation and methylthiolation, as well as the addition and removal of С-terminal amino-acid residues. However, while there is some evidence that certain modifications affect translational accuracy, or ribosome stability, in most cases their functional and physiological significance is unknown [7].

In *Escherichia coli*, the α-L-glutamate ligase RimK catalyzes the unique, C-terminal addition of glutamate residues to the ribosomal 30S subunit protein S6 (RpsF). The biochemistry of the transferase reaction [8] and the synthesis of poly-α-L-glutamate peptides [9] have been studied *in vitro* for *E*. *coli* RimK, and a crystal structure for this protein is available [10]. However, while the phenomenon of glutamate addition by RimK is clearly documented, the significance of this modification for ribosomal function and cell behavior remains unknown. The *rimK* gene is widespread, with homologs in hundreds of prokaryotic and eukaryotic genomes, so determining the biological role of RimK has broad implications for our understanding of ribosome structure and function. Recently, the *rim* locus (*PFLU0261-0263*) was identified as part of an *In Vivo Expression Technology* (IVET) screen for up-regulated loci during *Pseudomonas fluorescens* interaction with sugar beet [11], prompting us to investigate further.

*P*. *fluorescens* is a Gram negative, soil-dwelling bacterium that non-specifically colonizes plant rhizospheres. Here, it utilizes root exudates as a carbon source and protects the host plant by positively affecting health and nutrition, and exhibiting potent antifungal and other biocontrol capabilities [12–14]. Successful plant colonisation by *P*. *fluorescens* is a complex process that requires the coordinated regulation of phenotypes including motility, the production of attachment factors such as exo/lipopolysaccharides, and the deployment of secondary metabolites [14–16]. Plant-colonizing bacteria must adapt both membrane transport and primary metabolism to exploit the carbon and nitrogen resources exuded by plant roots. A recent study of *Rhizobium leguminosarum* rhizosphere transcriptomes showed both a metabolic shift towards the utilization of organic acids as the principle carbon source, and the up-regulation of ABC transporters for molecules including oligosaccharides and various amino acids [17].

The related species *P*. *syringae and P*. *aeruginosa* also encode the *rimABK* genes (*rimBK* only in *P*. *aeruginosa*). The phytopathogen *P*. *syringae* is responsible for a range of economically important plant diseases. It produces an array of species-specific type-III-secreted effectors and phytotoxins to subvert plant defences [18,19] and infects host plants by migration through open stomata and wounds on plant surfaces. It then colonizes the apoplastic space, multiplying rapidly and leading to chlorosis and tissue necrosis [18]. *P*. *aeruginosa* is an opportunistic pathogen of plants and humans, and the predominant infective bacterium in late stage cystic fibrosis lung infections [20]. It is a highly flexible pathogen, utilizing diverse phenotypic outputs to colonise and infect hosts including both plants and humans [21]. While responses to the environment by *P*. *aeruginosa* are complex, they may be broadly categorized as promoting either acute (virulent, cytotoxic and motile) or chronic (persistent, biofilm forming) lifestyles. Transitions between the two are frequently prompted by genetic adaptation during long-term infections [22].

In addition to *rimK* the *P*. *fluorescens rim* locus contains *rimB*, encoding a small uncharacterized protein, and *rimA*, which encodes an EAL (phosphodiesterase) domain for the ubiquitous bacterial second messenger cyclic-di-GMP (cdG). CdG signalling pathways control a wide range of processes involved in the transition between sessile, biofilm forming and unicellular, motile and virulent lifestyles in the majority of bacterial species [23,24]. In general, high cdG levels are associated with community behavior while low levels promote virulence and motility [23]. CdG influences bacterial phenotypes by binding and affecting the function of specific effector proteins, the identity of which can be difficult to predict in advance [25,26]. CdG signal transduction in *Pseudomonas* is highly complex, with dozens of metabolic proteins [27] and phenotypic outputs including exopolysaccharide and adhesin synthesis [28,29], virulence and cytotoxicity [27,30] and the production and control of flagella [28,31,32].

In this study we define the function of the RimABK system and its role in controlling interactions between *Pseudomonas* spp. and plants. Deletion of the *rimABK* genes down-regulates motility and virulence, and promotes phenotypes associated with sessile, surface-associated lifestyles in the commensal *P*. *fluorescens*, and the pathogens *P*. *syringae and P*. *aeruginosa*. RimK post-translationally modifies the ribosomal protein RpsF by the addition of glutamate residues to its C-terminus. This modification has profound effects on the *Pseudomonas* ribosome, with *rimK* deletion leading to significantly lower abundance of multiple ribosomal proteins, although the level of rRNA remains unaffected. Loss of modification by RimK manifests in specific changes in the proteome of *P*. *fluorescens*. These include a marked reduction in the important translational regulator Hfq and corresponding increases in non-ribosomal peptide synthetases (NRPS), stress response proteins and ABC transporters for peptides, polyamines and amino acids. RimK activity appears to be tightly controlled, both transcriptionally and via direct interaction with RimA, RimB and cdG, with addition of all three stimulating RimK enzymatic activity *in vitro*. We propose that control of RimK ribosomal modification represents a novel, high-level regulatory mechanism that enables bacteria to ‘fine-tune’ their proteomes to appropriately respond to the surrounding environment.

## Results

### RimABK influences *P*. *fluorescens* rhizosphere colonisation, root attachment and swarming motility

**Fig 1:**
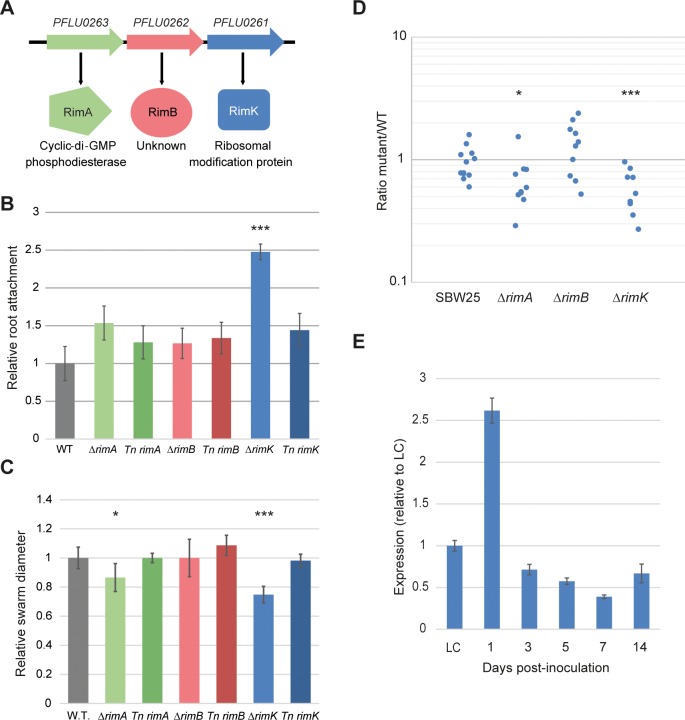
RimABK is important for *P*. *fluorescens* rhizosphere colonisation. **1A.** The SBW25 *rimABK* operon consists of three co-transcribed genes. Gene numbers and predicted translated proteins are shown in each case. **1B.** Wheat root attachment by the Δ*rimABK* and complementation strains relative to SBW25 WT. **1C.** Swarming motility of the Δ*rimABK* and complementation strains relative to SBW25 WT. **1D.** Rhizosphere colonisation competition assays. The graph shows the ratio of SBW25 WT or Δ*rimABK* to WT-*lacZ* colony forming units (CFU) recovered from the rhizospheres of wheat plants seven days post-inoculation. Each dot represents CFU recovered from an individual plant. Statistically significant differences between SBW25 and Δ*rimABK* strains are indicated (*** = p < 0.01, * = p < 0.05) in each case. **1E.** SBW25 *rimK* mRNA abundance determined by qRT-PCR, for wheat rhizospheres sampled at various intervals post-inoculation. Expression of *rimK* is shown relative to M9 0.4% pyruvate liquid-culture (LC).

The *P*. *fluorescens* SBW25 *rimABK* locus is a predicted three gene polycistronic operon (Fig 1A). Following the observation that the *rim* locus is up-regulated in the plant environment [11] we first examined how the three *rim* genes affect different phenotypes associated with plant interaction. To do this, we deleted the *rim* genes, and confirmed that neither of the two upstream deletions (*rimA/B*) had significant effects on transcription of the third gene, *rimK* (S1A Fig). Next, we complemented each deletion with a chromosomally-inserted copy under the predicted *rim* promoter at the *att*::Tn*7* locus [33]. Deletion of *rimK*, and to a lesser extent *rimA* led to enhanced wheat root attachment relative to WT SBW25 (Fig 1B), and also to a defect in swarming motility (Fig 1C). Both phenotypes were complemented in the relevant Tn*7* strain. To test the importance of *rimABK* for growth in the rhizosphere environment, we next examined the ability of the *rim* mutants to competitively colonise the rhizospheres of wheat seedlings. After seven days, significantly fewer Δ*rimK* and Δ*rimA* colony forming units (CFUs) were recovered from model rhizospheres compared with the WT-*lacZ* competitor (Fig 1D). No differences in growth-rate were seen for the Δ*rimABK* mutants compared with WT in rich or poor defined media (S1B and S1C Fig), suggesting that the observed colonisation defects are specific to the rhizosphere environment. Finally, to test whether *rim* transcription varies as the surrounding environment changes, we examined *rimK* mRNA abundance at different points during wheat rhizosphere colonisation. SBW25 mRNA was extracted from inoculated wheat rhizospheres after incubation periods of between 1 and 14 days, and *rimK* mRNA levels assayed by qRT-PCR. 1-day colonisation samples showed significantly increased (261.7 ± 14.8%) transcript abundance compared with overnight growth in M9 pyruvate. However, *rimK* expression then decreased sharply as colonisation proceeded, with mRNA abundance after 7 days at 39.1 ± 2.1% of that seen in liquid-culture (Fig 1E), representing an almost seven-fold drop from the levels seen in day 1. This suggests that *rimABK* expression may be triggered by signals present in the early wheat rhizosphere, then down-regulated in the established root environment.

### RimK also controls motility, attachment and virulence in plant and human pathogens

**Fig 2:**
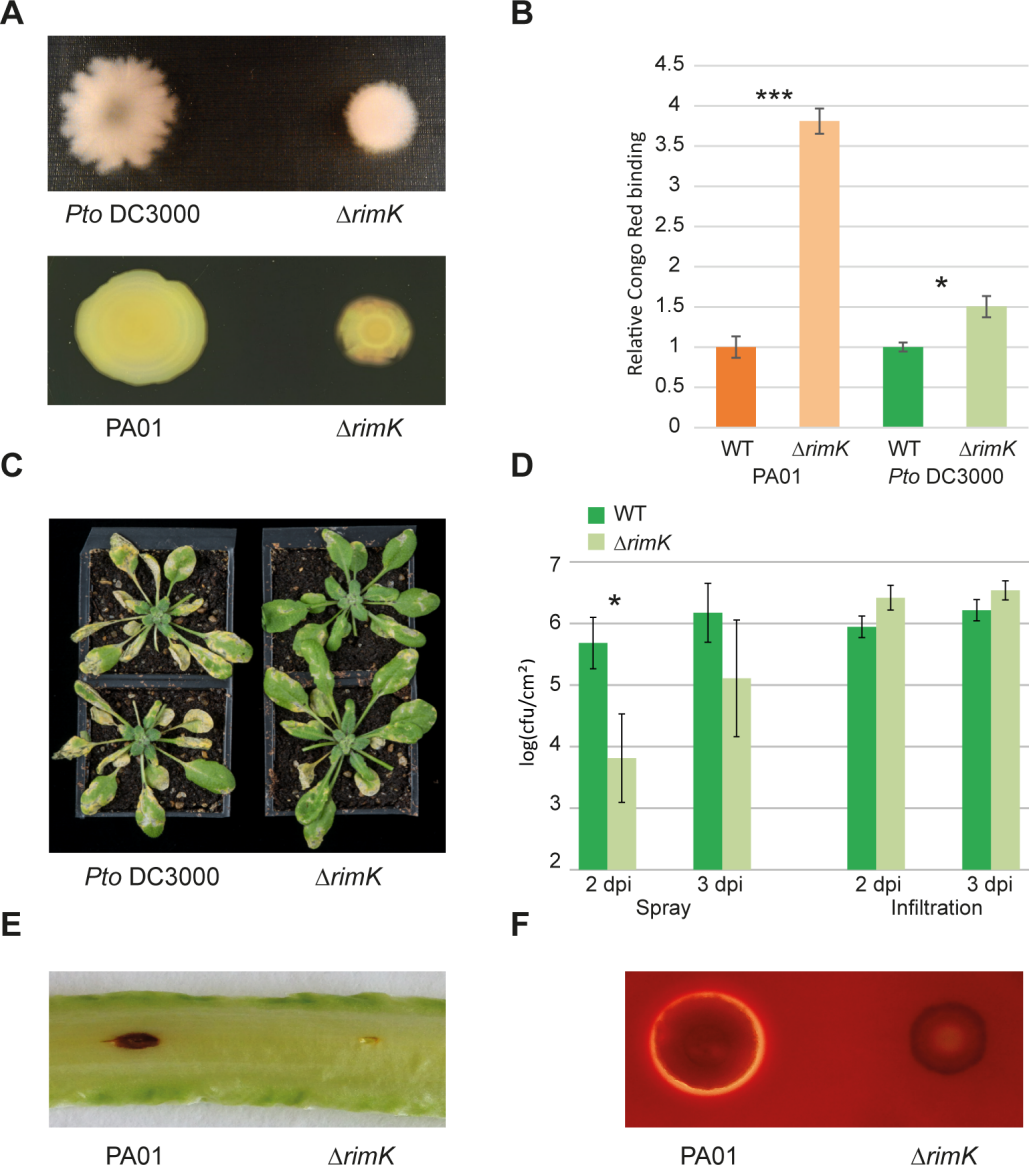
RimK is important for *P*. *syringae* and *P*. *aeruginosa* plant infection. **2A.** Swarming motility of *Pto* DC3000 and PA01 Δ*rimK* relative to their respective WT strains. **2B.** Congo Red binding of *Pto* DC3000 and PA01 Δ*rimK* compared with their respective WT strains. **2C.** Representative spray-infected *Arabidopsis* Col-0 plants 4 days post-infection with *Pto* DC3000 WT/Δ*rimK*. Disease symptoms are less marked with Δ*rimK* infection. **2D.** log (*P*. *syringae* CFU per cm^2^ leaf tissue) recovered from *Arabidopsis* Col-0 plants infected with *Pto* DC3000 WT or Δ*rimK*, 2 and 3 days post-infection (dpi). The infection method in each case is stated beneath the graph. **2E.** Lettuce leaf infections with *P*. *aeruginosa* WT/Δ*rimK* strains. Lesions photographed after 5 days. **2F.** β-hemolysis by *P*. *aeruginosa* WT/Δ*rimK* strains after 24 h growth on horse blood agar.

To examine whether RimK affects the plant-association behavior of related, pathogenic *Pseudomonas* species, we investigated the impact of *rimK* deletion in the phytopathogen *P*. *syringae* pv. *tomato* (*Pto*) DC3000 [34] and the opportunistic human pathogen *P*. *aeruginosa* PA01. Consistent with the findings for SBW25, deletion of *rimK* in *Pto* DC3000 led to increased Congo Red (CR) binding (an assay for lipo/exopolysaccharides and proteinaceous attachment factors [35]) and reduced swarming motility compared to WT (Fig 2A and 2B). Next, we examined the effect of *rimK* deletion on *Pto* DC3000 infection of *Arabidopsis thaliana* Col-0. Upon spray infection, Δ*rimK* presented both noticeably milder disease symptoms (Fig 2C) and significantly reduced bacterial proliferation in the apoplast. In contrast, no differences were observed between WT and Δ*rimK* in a leaf infiltration assay (Fig 2D), suggesting that *rimK* is important during the early stages of *P*. *syringae* plant infection only. Compared to WT PA01, Δ*rimK* also showed reduced swarming motility (Fig 2A) and significantly increased CR binding (Fig 2B). Furthermore, PA01 Δ*rimK* was markedly compromised both in its ability to infect lettuce leaves [36] and to induce β-hemolysis on blood agar plates (Fig 2E and 2F), while growth in liquid media was unaffected (S1D Fig).

### RimK interacts with the ribosome and affects its function

**Fig 3:**
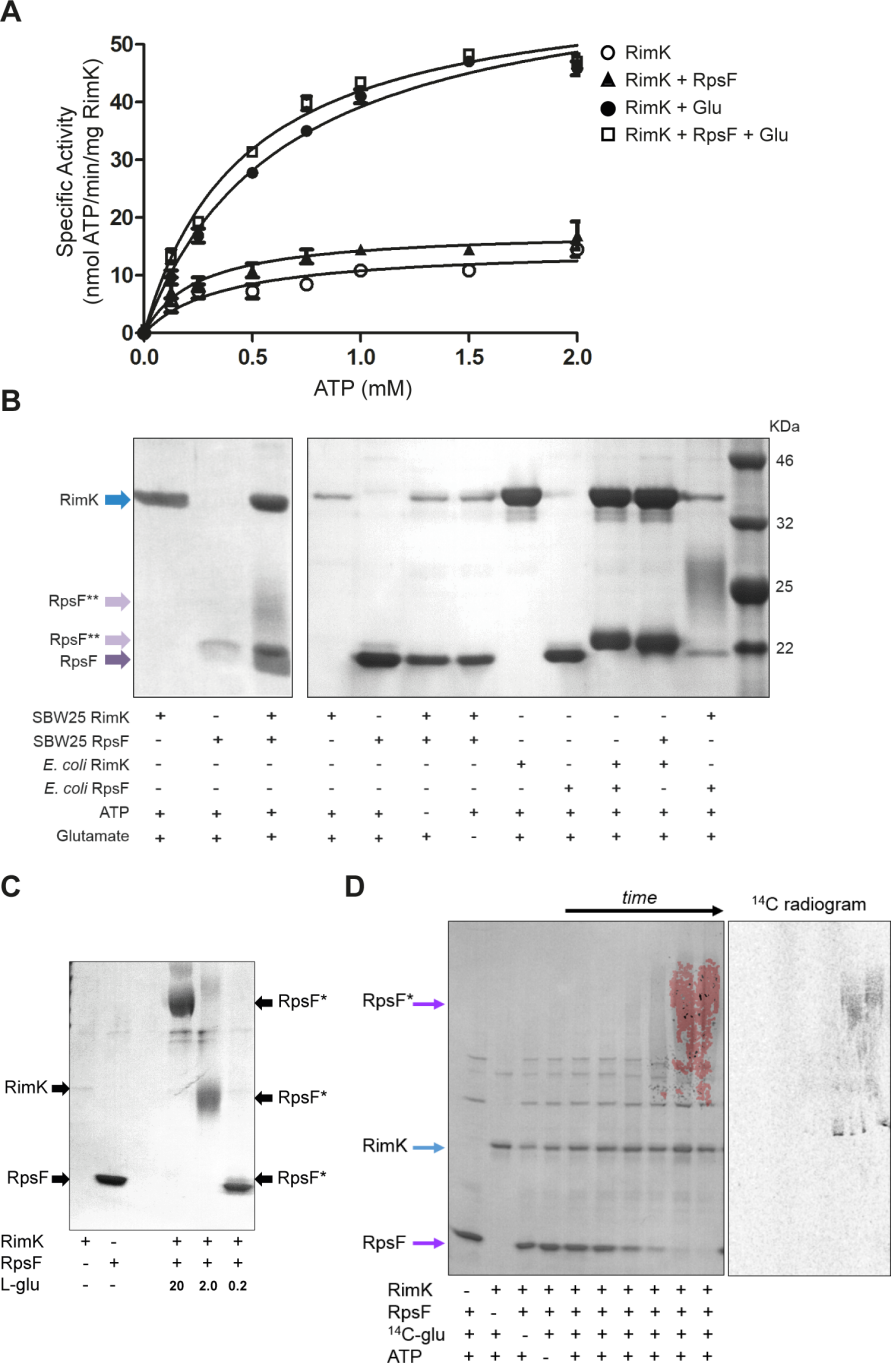
Biochemical analysis of RimK. **3A.** ATPase activity of RimK_Pf_ incubated with RpsF_Pf_ and glutamate. RimK_Pf_ specific activity (nmol ATP hydrolyzed/min/mg RimK_Pf_) is shown for increasing concentrations of ATP (open circles). Addition of RpsF_Pf_ (triangles), glutamate (filled circles) or both (square) increases the *V*_*max*_ of RimK_Pf_ ATPase activity. **3B.** Glutamation assays with *E*. *coli* and SBW25 RimK and RpsF. The contents of each assay are indicated underneath the relevant lanes. Independent preparations of RimK_Pf_ and RpsF_Pf_ were used in the two panels, which were run separately and are shown side by side for comparative purposes only. Running positions of RimK, RpsF and glutamated-RpsF (RpsF**) are marked with arrows. **3C.** Glutamation assays with RimK_Pf_ and RpsF_Pf_. The contents of each assay are indicated, with 0.2, 2.0 and 20 mM glutamate added to the test samples as shown. Running positions of RimK, RpsF and glutamated-RpsF (RpsF*) are marked. **3D.** Glutamation assays with RimK_Pf_, RpsF_Pf_ and U-^14^C- glutamate. The contents of each reaction is indicated underneath the relevant lane. Control samples were incubated overnight, while time-course samples show 5, 10, 30, 60, 180 minutes, and overnight incubation. The left hand panel shows an overlay of Coomassie stained and radiolabel visualizations of a single gel. The right hand panel shows radiolabel incorporation into RpsF alone.

*E*. *coli* RimK is an α-l-glutamate ligase and catalyzes both the ATP-dependent synthesis of poly-α-l-glutamate peptides [9], and the sequential addition of glutamate residues to the C-terminus of ribosomal protein RpsF [8]. To examine the relationship between these two proteins in *P*. *fluorescens*, SBW25 RimK and RpsF (RimK_Pf_/RpsF_Pf_) were purified and used to test the parameters of RimK enzyme activity. First, a linked pyruvate kinase-lactate dehydrogenase (PK/LDH) assay was used to test RimK_Pf_ ATPase activity (Fig 3A). Alone, SBW25 RimK displayed low-level ATPase activity (*K*_*m*_ 0.37 ± 0.13 mM, *V*_*max*_ 14.84 ± 1.6 nmol/min/mg protein). The *V*_*max*_ of RimK_Pf_ barely increased with RpsF_Pf_ (to 17.8 ± 1.3 nmol/min/mg), but changed more noticeably upon glutamate addition (to 64.3 ± 2.8 nmol/min/mg). While *K*_*m*_ did not change markedly with the addition of either co-factor, *V*_*max*_*/K*_*m*_ increased steadily (RimK = 39.7, +RpsF = 71, +Glu = 100.3, +both = 132.7), an indication of increasing enzymatic efficiency.

Next, we examined RpsF_Pf_ glutamation using SDS-PAGE gel-shift assays adapted from Kino *et al*. [9]. Purified RimK_Pf_ increased the mass of RpsF_Pf_ (Fig 3B), consistent with previous observations for the addition of C-terminal glutamate residues [9]. MALDI-TOF analysis confirmed that the shifted band was overwhelmingly composed of RpsF peptides, although we were unable to detect the glutamated C-terminal RpsF peptide in this sample. This may be due to its large size (>3000 Da) and strong negative charge resulting in a failure to be retained on the reverse phase column. To examine the relationship between RimK and RpsF in more detail, we purified the *E*. *coli* homologs of both proteins (RimK_Ec_/RpsF_Ec_) and used these to carry out additional gel-shift experiments (Fig 3B). Unlike RimK_Pf_, ATPase activity for *E*. *coli* RimK was strictly glutamate dependent, with no activity seen for RimK_Ec_ alone (S2 Fig). As expected, purified RimK_Ec_ increased the mass of RpsF_Ec_ by adding glutamate residues to its C-terminus [9]. Interestingly, the RimK proteins from *E*. *coli* and SBW25 were functionally interchangeable: RimK_Ec_ was also able to shift RpsF_Pf_, and RimK_Pf_ successfully increased the mass of RpsF_Ec_ (Fig 3B). The activity of both RimK homologs to modify RpsF was strictly dependent on the presence of glutamate and ATP in the reaction mix.

While we did occasionally see a conventional RpsF band-shift (Fig 3B), in many cases RimK_Pf_ activity led to the formation of very large, diffuse RpsF bands (confirmed by MALDI-TOF) that ran poorly in SDS-PAGE. Formation of these large, diffuse bands was strictly dependent on the glutamate concentration in the assay, with lower levels producing more conventional band shifts (Fig 3C), and suggesting that the hyper-shifting band pattern seen with RimK_Pf_ is due to uncontrolled RpsF glutamation in our assay conditions. To confirm that the RpsF in the large, diffuse complexes was indeed glutamated, gel-shift assays were repeated with radiolabeled ^14^C-glutamate. Progressive incorporation of radiolabel into the RpsF band was seen over 24 hours (Fig 3D), confirming that RimK_Pf_ functions as an α-l-glutamate ligase of RpsF.

To further investigate the effects of RpsF glutamation by RimK on the ribosome, we first examined the effect of *rimK* deletion on ribosomal RNA abundance by qRT-PCR of the 16S rRNA. No significant differences were observed between WT SBW25 and the *rimK* deletion mutant (Δ*rimK*/WT 16S rRNA = 1.27 ± 0.1). Next, we purified ribosomes from SBW25 WT and Δ*rimK* using sucrose cushion ultracentrifugation and examined the relative abundance of ribosomal proteins by semi-quantitative, comparative LFQ analysis using MaxQuant software. Strikingly, almost every detected ribosomal protein was present at a considerably lower level in Δ*rimK* compared with WT (S1 Table), despite their similar 16S rRNA levels. Consistent with a role for RimK in the regulation of ribosome function, *rimK* overexpression significantly increased SBW25 sensitivity to the 30S-targeting aminoglycosides gentamycin and kanamycin, but had no effect on sensitivity to the MurA inhibitor phosphomycin (S3 Fig). This suggests that excess RimK activity affects the ribosome in particular, rather than conferring a non-specific sensitivity to antibiotics in general.

### RimK activity is controlled by RimA, RimB and the dinucleotide second messenger cdG

Next, we probed the protein-protein interactions of RimABK by co-immunoprecipitation with flag-tagged proteins. As expected, the RimK assay highlighted potential interactions with multiple ribosomally-associated proteins (Table 1), consistent with a role for RimK in ribosomal modification. Interestingly, both RimB and RimA also pulled down similar numbers of ribosomal protein peptides, alongside multiple peptides from the other two Rim proteins. These data suggest that the RimABK proteins associate both with each other and with the ribosome, and posit a role for RimA and RimB in the regulation of RimK activity.

**Table 1:** RimK interacting proteins. Numbers denote unique peptides detected in each sample.

	RimK	RimB	RimA	Negative Ctrl	Positive Ctrl
**Ribosomal protein S6 modification protein RimK**	**95**	17	10	0	0
**Putative uncharacterized protein RimB**	0	**46**	9	0	0
**Conserved EAL domain protein RimA**	0	0	**55**	0	0
**50S ribosomal protein L18**	7	7	6	1	2
Putative glycosyl transferase PFLU0478	7	4	0	0	0
**50S ribosomal protein L2**	6	12	2	0	3
Lon protease PFLU3927	5	4	2	0	0
**30S ribosomal protein S13**	5	4	1	0	2
**Translation initiation factor IF-2**	3	12	1	0	0
**50S ribosomal protein L20**	3	3	0	0	0
UPF0229 protein PFLU5583	3	1	2	0	0
Chorismate synthase AroC	1	11	0	0	0
Putative sulfite reductase PFLU2657	0	7	0	0	0
Putative peptidase PFLU2126	1	6	0	0	0
Putative uncharacterized protein PFLU4307	0	6	0	0	0
DNA-directed RNA polymerase subunit beta RpoB	0	4	1	0	0
**30S ribosomal protein S21**	0	4	0	1	0
**30S ribosomal protein S11**	1	3	1	0	0
**30S ribosomal protein S12**	1	3	0	0	0
Chaperone ClpB2	1	1	14	0	4
Cell division protein FtsA	0	1	12	0	2
Alanine—tRNA ligase AlaS	1	1	11	0	3
ATP-dependent Clp protease ATP-binding subunit ClpX	1	0	11	0	1
Putative carbamoyltransferase PFLU0475	0	0	7	0	0
Glycine—tRNA ligase alpha subunit GlyQ	0	0	7	0	0
Ribonucleoside-diphosphate reductase β-chain PFLU4768	0	0	6	0	0
Short chain dehydrogenase PFLU3332	0	0	6	0	0
2-dehydropantoate 2-reductase PFLU1707	0	0	6	0	0
Glutamate-ammonia-ligase adenylyl transferase GlnE	0	0	6	0	0
Putative uncharacterized protein PFLU1879	0	0	5	0	0
Putative uncharacterized protein PFLU5584	0	0	5	0	0
Putative histidine lyase PFLU0366	0	0	5	0	0

**Fig 4:**
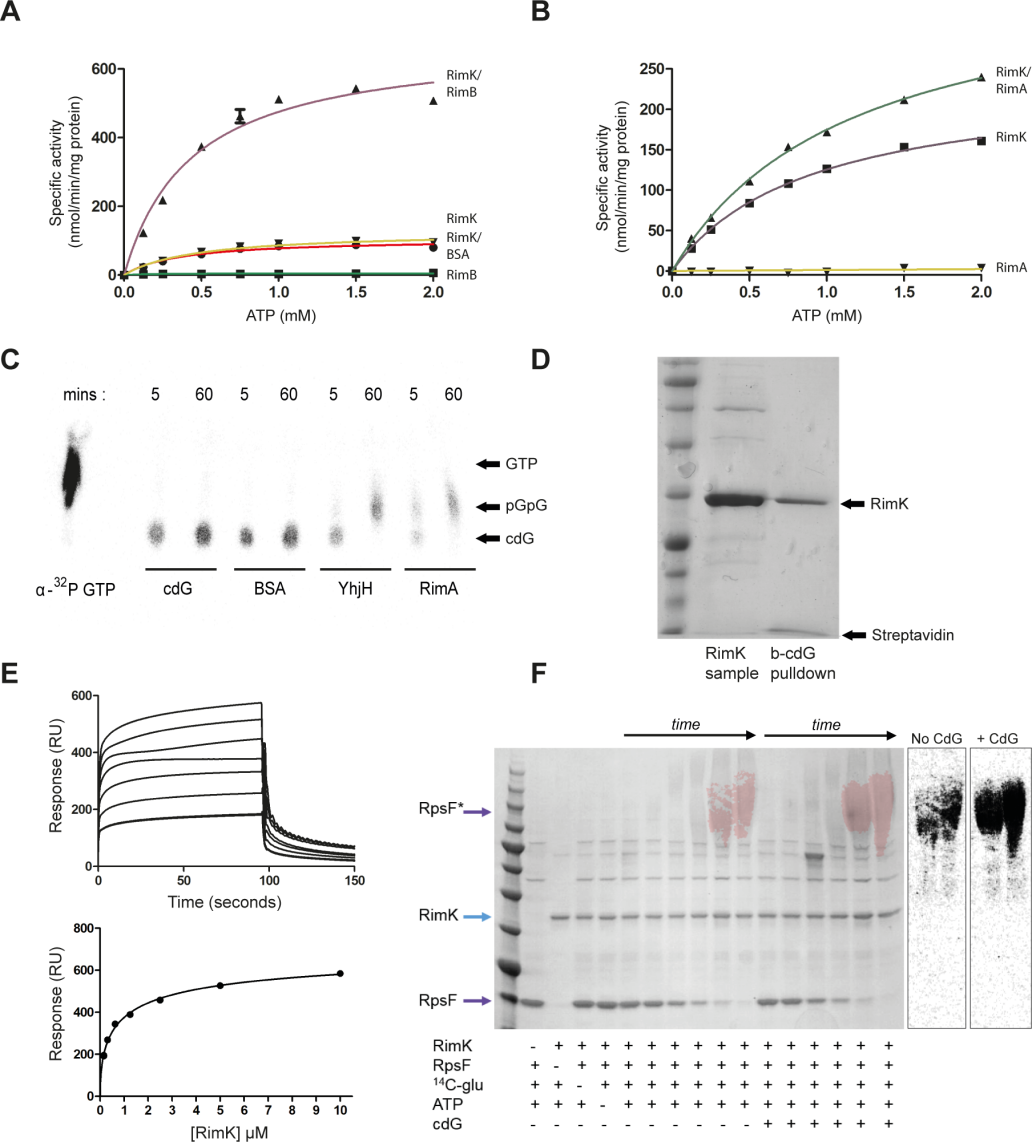
RimA, RimB and cdG impacts on RimK activity. **4A.** ATPase activity of RimK_Pf_ incubated with RimB, BSA and glutamate. RimK_Pf_ specific activity (nmol ATP hydrolyzed/min/mg RimK_Pf_) is shown for increasing concentrations of ATP (red, circles). Addition of RimB (purple, up-triangles) increases the RimK_Pf_ *V*_*max*_, while BSA (brown, down-triangles) does not. RimB alone displays no ATPase activity (green, squares). **4B.** ATPase activity of RimK_Pf_ incubated with RimA and glutamate. RimK_Pf_ specific activity (nmol ATP hydrolyzed/min/mg RimK_Pf_) is shown for increasing concentrations of ATP (purple, squares). Addition of RimA (green, up-triangles) increases the RimK_Pf_ *V*_*max*_, while RimA alone displays no ATPase activity (yellow, down-triangles). **4C.** Thin layer chromatography of α-^32^P-labeled cdG incubated with BSA, YhjH or RimA for the time periods shown. The product of cdG hydrolysis; pGpG, migrates further than cdG but less than α-^32^P-GTP. **4D.** Biotinylated-cdG pulldown for RimK_Pf_. *E*. *coli* overexpression cell lysate (RimK sample) is loaded alongside the washed cdG-bead sample (b-cdG pulldown). RimK and streptavidin are indicated with arrows. **4E.** SPR sensorgram and affinity data for RimK_Pf_ binding to biotinylated cdG. A range of RimK_Pf_ concentrations was used (0.156, 0.312, 0.625, 1.25, 2.5, 5, and 10 μM) and concentration replicates included as appropriate together with buffer only controls. Protein binding and dissociation phases are shown. For the affinity fit, binding responses were measured 4s before the end of the injection and *K*_*d*_ values for each protein calculated using BiaEvaluation software and confirmed by GraphPad. **4F.** The effect of cdG addition on glutamation of RpsF_Pf_ by RimK_Pf_. The contents of each reaction is indicated underneath the relevant lanes. Control samples were incubated overnight, while time-course samples show 5, 10, 30, 60, 180 minutes, and overnight incubation. The panel shows an overlay of Coomassie staining and radiolabel visualization (red) of the same gel, as with Fig 3C.

To test this, RimK_Pf_ ATPase assays were repeated with the addition of purified RimA/RimB proteins. Addition of RimB led to a substantial increase in RimK_Pf_ activity (e.g. *V*_*max*_ = 691.2 nmol/min/mg, 113.6 without RimB, Fig 4A), while RimA addition produced a consistent, but much smaller activity increase (e.g. *V*_*max*_ = 363.6 nmol/min/mg, 234.9 without RimA, Fig 4B). Again, increases in *V*_*max*_ were accompanied by corresponding enzyme efficiency increases. The modest effect of RimA was puzzling given the relative phenotypic effects of *rimA/rimB* disruption, and suggested another function for RimA besides direct stimulatory interaction with RimK. RimA contains an EAL domain, and is predicted to function as a phosphodiesterase (PDE) for the second messenger cdG. RimA PDE activity was subsequently confirmed (Fig 4C) using a chromatography-based assay alongside an established PDE; YhjH from *E*. *coli* [37]. This activity presented the intriguing possibility that cdG may play a role in the regulation of RimK activity, prompting us to test the relationship between cdG and RimK. Excitingly, both RimK_Pf_ and RimK_Ec_ were shown to bind strongly to cdG in biotinylated cdG pull-down assays and with surface plasmon resonance (SPR) after [38], with *K*_*d*_ values of 1.0 and 3.8 μM respectively (Fig 4D and 4E, and S4A Fig). Furthermore, cdG addition was shown to substantially increase both RimK_Pf_ ATPase activity (S4B Fig) and the amount of radiolabeled glutamate incorporated into RpsF *in vitro* (to 225 ± 59% of the cdG- sample, Fig 4E). These data establish a role for cdG signalling in the direct control of RimK activity, and hence ribosome modification.

### Deletion of *rimK* induces specific changes in the *P*. *fluorescens* proteome

**Fig 5:**
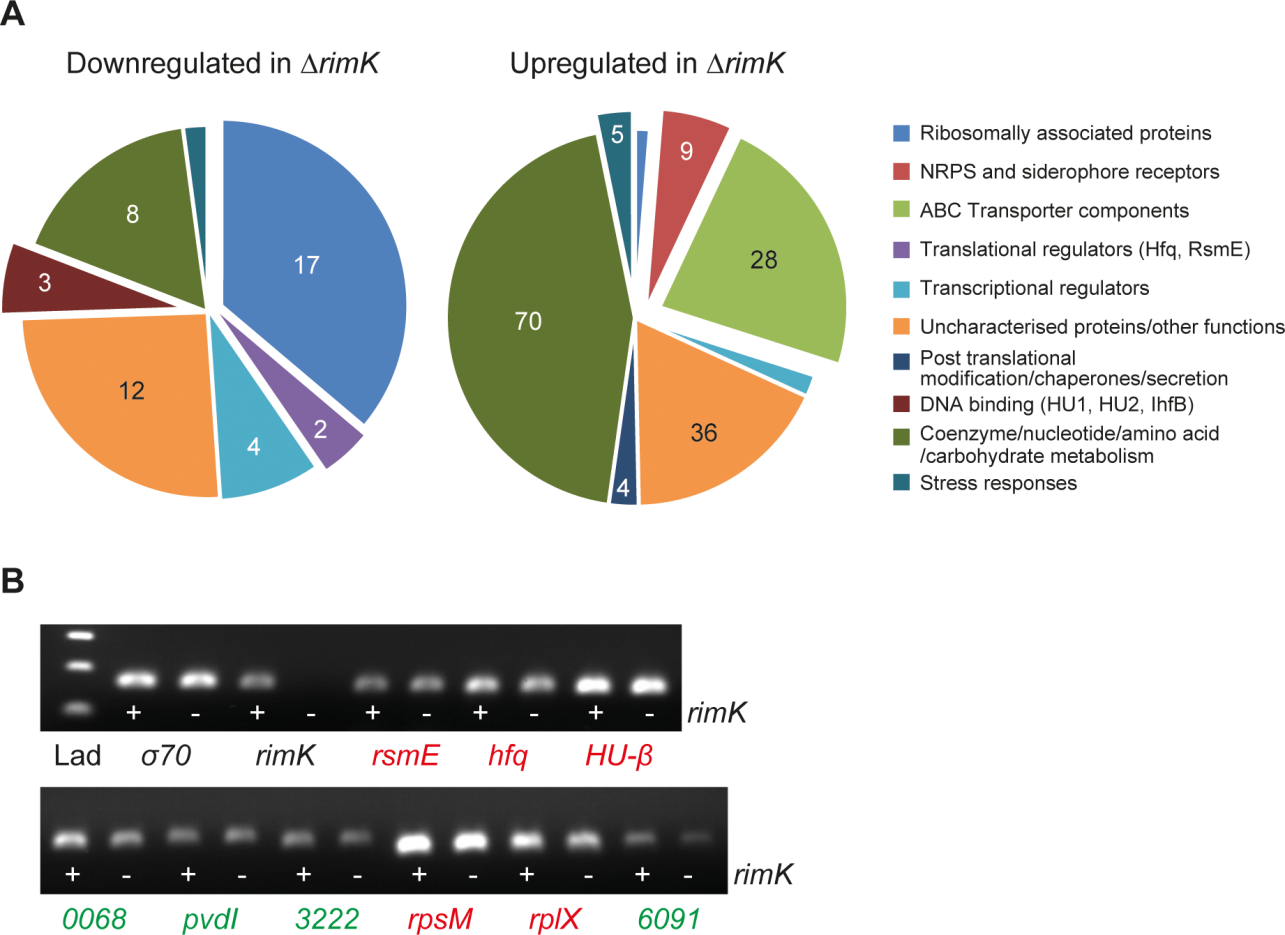
The RimK regulon in *Pseudomonas fluorescens*. **5A.** Up and down-regulated proteins in the Δ*rimK* mutant compared with SBW25 WT. Pie chart sections indicate the proportion of significantly up- or down-regulated proteins in each functional category as shown. The numbers in each section of the chart refer to the total number of proteins in that category. Interesting or important functional groups are expanded from the chart in each case. **5B.** RT-PCR showing relative mRNA abundance in SBW25 WT (+) or Δ*rimK* (-) for selected RimK-regulated proteins. The housekeeping gene *rpoD* (σ^70^) and *rimK* are included as controls. Down-regulated proteins in Δ*rimK* are shown in red, up-regulated proteins in green. (*PFLU*)*-0068* and *6091* encode ABC transporter components, *3222* an NRPS subunit.

To more closely examine how RimK affects ribosomal behaviour *in vivo*, and to probe the wider effects of this on the bacterial proteome, global quantitative mass-spectrometry analysis was conducted on soluble proteomes from liquid-culture grown SBW25 WT and Δ*rimK*. Approximately 1,000 proteins were identified in all tested proteomes (S6 Table, 2 biological replicates of WT and Δ*rimK*), of which 47 were significantly down-regulated and 157 up-regulated in Δ*rimK* across two independent experiments (Fig 5A, S2 Table). Once again, reduced levels of 17 ribosomal proteins were detected in the Δ*rimK* mutant relative to WT, alongside (possibly compensatory) increases in levels of elongation factor P (EF-P) and ribosome-recycling factor (Frr).

One of the most strongly down-regulated proteins in the Δ*rimK* proteome was the global translational regulator Hfq (Fig 5A, Table 2). Hfq is a small, hexameric RNA-binding protein that exerts pleiotropic effects on mRNA translation by mechanisms that include facilitating the binding of regulatory sRNAs with their mRNA targets [5,39] and targeting the degradation of selected mRNAs [40–42]. Hfq may also act as a direct repressor of mRNA translation [43]. Δ*rimK* also contained markedly increased levels of numerous ABC transporter subunits, primarily those for amino acids, dipeptides and the polyamine putrescine. Several ABC-exported peptides were also strongly up-regulated (Table 2). Altered ABC transporter abundance has been linked to *hfq* deletion in several studies [44–46].

**Table 2:** Up- and Down-regulated proteins in the Δ*rimK* mutant background.

***Down-regulated in mutant***	***ΔrimK fold-change***		
	**Assay 1**	**Assay 2**	**Average**
Putative aldolase PFLU_4292	-12.84	-14.24	-13.54
Protein Hfq	-7.54	-2.92	-5.23
Putative D-hydantoinase PFLU_3942	-3.91	-5.39	-4.65
50S ribosomal protein L34 rpmH	-6.36	-2.06	-4.21
50S ribosomal protein L24 rplX	-5.28	-2.70	-3.99
Putative uncharacterized protein PFLU_1522	-5.00	-2.57	-3.78
Predicted RNA-binding protein, possibly ribosomal protein PFLU_5262	-5.36	-1.88	-3.62
Putative uncharacterized protein PFLU_2951	-3.46	-3.44	-3.45
Putative outer membrane protein PFLU_1327	-3.82	-2.63	-3.22
Putative uncharacterized protein PFLU_3617	-3.75	-2.26	-3.00
50S ribosomal protein L16 rplP	-3.57	-1.97	-2.77
Putative regulatory protein PFLU_2993	-3.40	-1.93	-2.66
30S ribosomal protein S15 rpsO	-3.65	-1.64	-2.64
Phosphate starvation-inducible protein psiF	-2.09	-3.16	-2.62
Transcriptional regulatory protein AlgP2	-2.91	-2.13	-2.52
Regulator of secondary metabolism RsmE PFLU_4165	-3.84	-1.18	-2.51
Transcriptional regulatory protein AlgP1 PFLU_5927	-2.81	-2.22	-2.51
DNA-binding protein HU1 hupA	-3.26	-1.64	-2.45
Ubiquinol-cytochrome c reductase iron-sulphur subunit petA	-2.95	-1.95	-2.45
30S ribosomal protein S13 rpsM	-2.92	-1.98	-2.45
Putative uncharacterized protein PFLU_5073	-3.02	-1.83	-2.42
30S ribosomal protein S21 rpsU	-3.22	-1.54	-2.38
Catalase katE	-1.63	-3.11	-2.37
2-nonaprenyl-3-methyl-6-methoxy-1,4-benzoquinol hydroxylase coq7	-2.40	-2.31	-2.36
Putative uncharacterized protein PFLU_0413	-3.43	-1.27	-2.35
***Up-regulated in mutant***	**Assay 1**	**Assay 2**	**Average**
Putative non-ribosomal peptide synthetase PFLU_3225	7.46	2.24	4.85
Putative amino-acid ABC transport system, substrate-binding protein PFLU_1000	5.49	4.29	4.89
Putative membrane protein PFLU_4501	5.85	4.63	5.24
Putative pyoverdin synthetase F PFLU_2547	5.65	4.83	5.24
Putative hydrolase PFLU_1856	6.71	3.82	5.26
Glutamate/aspartate ABC transport system, periplasmic binding protein gltI	6.45	4.17	5.31
Pyoverdin synthetase J pvdJ	7.75	2.93	5.34
Putative histidine-binding periplasmic protein PFLU_4765	5.65	5.35	5.50
Putative ABC transport system, exported protein PFLU_0246	5.75	5.26	5.51
Putative aminotransferase PFLU_5135	9.52	1.64	5.58
Putative uncharacterized protein PFLU_3504	9.52	1.66	5.59
Putative amino-acid transport system, substrate-binding protein PFLU_1311	6.10	5.29	5.69
Putative rhizopine-binding ABC transporter protein PFLU_2583	5.85	5.59	5.72
Aromatic-amino-acid aminotransferase PFLU_4209	8.00	3.73	5.87
Putative ubiquinol—cytochrome C reductase, cytochrome C1 PFLU_0843	9.26	3.00	6.13
Biopolymer transport membrane protein exbB	9.43	3.12	6.27
Putative branched amino-acid ABC transport system, substrate-binding protein livJ2	6.67	6.02	6.35
Dipeptide ABC transport system, substrate-binding protein dppA2	8.26	4.44	6.35
Putative amino-acid ABC transport system, membrane protein PFLU_0313	6.80	6.21	6.51
Putrescine ABC transport system, substrate-binding periplasmic protein potF1	6.67	6.45	6.56
Putative exported protein PFLU_0215	5.41	7.87	6.64
Glucose-6-phosphate isomerase pgi	12.20	1.78	6.99
Diaminobutyrate—2-oxoglutarate aminotransferase PFLU_4378	7.52	6.62	7.07
Putative D-methionine ABC transport system, substrate-binding protein PFLU_0068	7.58	6.62	7.10
Dipeptide ABC transport system, substrate-binding protein dppA3	9.35	5.52	7.44
Branched amino-acid ABC transport system, substrate-binding protein livJ1	8.85	7.09	7.97
Preprotein translocase subunit PFLU_5076	13.89	2.29	8.09
Molybdate-binding periplasmic protein PFLU_2971	9.26	7.04	8.15
Pyoverdin synthetase I pvdI	12.82	3.53	8.18
Aspartate-semialdehyde dehydrogenase asd	11.76	4.90	8.33
UPF0312 protein PFLU_5725	8.06	8.62	8.34
Putative membrane protein PFLU_0832	14.71	4.35	9.53
Putative ABC transport system, periplasmic protein PFLU_6091	8.70	19.23	13.96

Seven NRPS genes, including three synthases for the iron scavenging siderophore pyoverdin (Pvd) were also significantly up-regulated in the Δ*rimK* strain. Hfq has been implicated in the control of iron homeostasis [44], again suggesting a link between these proteome changes and Hfq down-regulation. A further important class of Δ*rimK* up-regulated proteins are those involved in oxidative stress responses, including superoxide dismutase (SodA), thioredoxin and glutathione S-transferase and reductase [47]. Once again, regulation of these proteins has been linked to Hfq [48]. Also up-regulated were several enzymes involved in the secretion and post-translational modification of proteins such as the isomerase SurA, disulfide oxidoreductase DsbA and the pre-protein translocase YajC. The up-regulation of these proteins is consistent with a role in processing and folding the excess periplasmic ABC transporter peptides found in the Δ*rimK* background.

In addition to the drop in Hfq levels, a smaller decrease was observed for a second translational regulator, RsmE [6]. Rsm family proteins control phenotypes including virulence, motility, exopolysaccharide production, carbon metabolism and stress responses in numerous Gram-negative bacteria [49–51]. Reduced protein abundance was also seen for the chromatin organization and DNA bending proteins HU1, HU-beta, and IhfB [52,53] (S2 Table). Several transcriptional regulators, including AlgP1 and AlgP2 [54,55] were down-regulated, while the osmosis regulator OmpR was significantly more abundant in the Δ*rimK* background (S2 Table). Clearly, the proteomic changes that arise as a consequence of *rimK* deletion are both complex and pleiotropic, and determining their relevance and interconnection is the subject of active enquiry. Following the proteomic analysis, RT-PCR was used to examine the relative mRNA abundance of nine significantly up/down-regulated proteins in WT and Δ*rimK*. No discernable change was observed in the mRNA levels of *hfq*, *rsmE*, or any of the other RimK up- or down-regulated proteins tested (Fig 5B), supporting the hypothesis that the observed differences between the Δ*rimK* and WT proteomes predominantly occur post-mRNA transcription.

### Downstream genetic analysis of the RimK regulon

**Fig 6:**
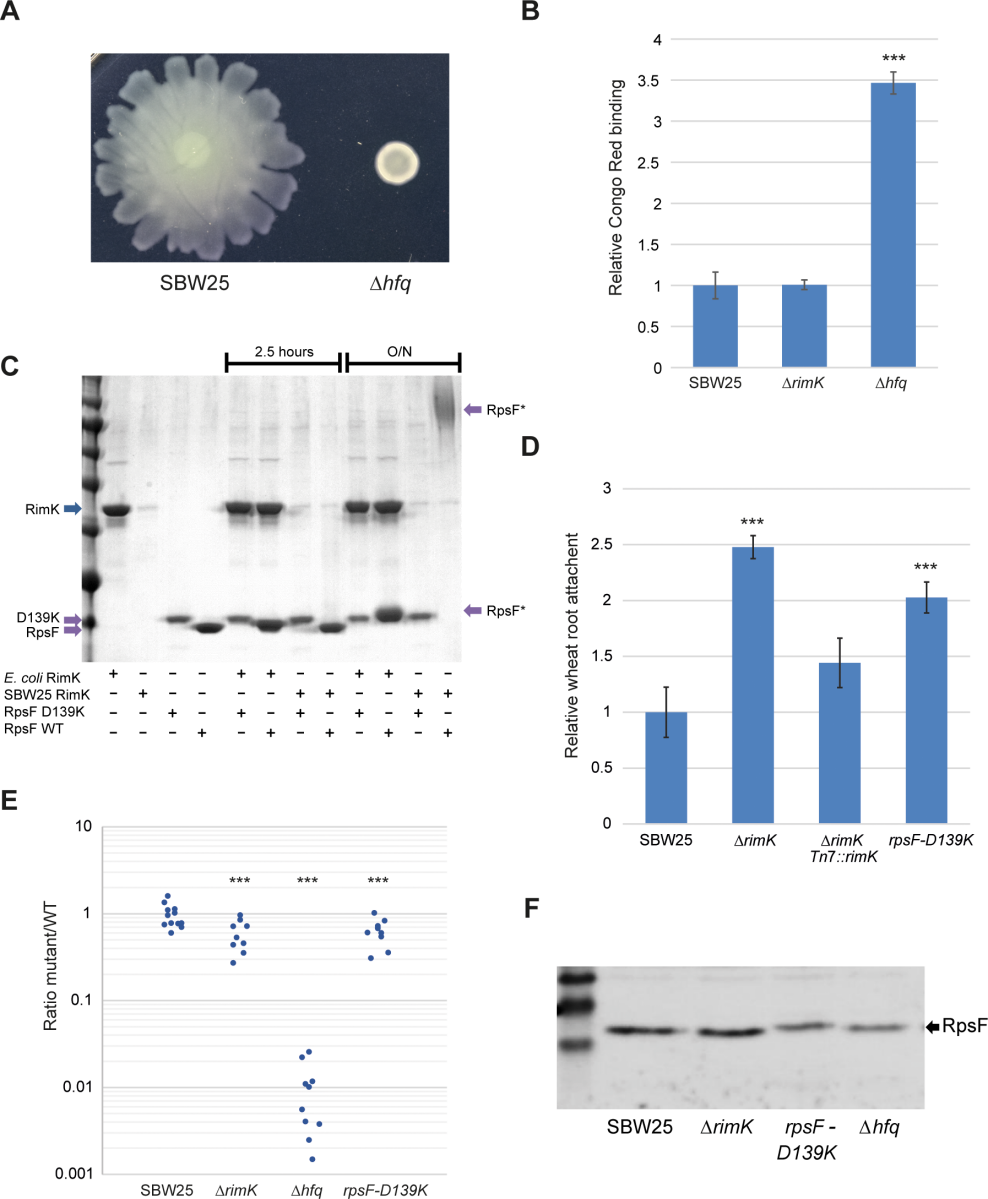
Comparison of the *P*. *fluorescens* Δ*rimK*, Δ*hfq* and *rpsF-D139K* mutant strains. **6A.** Swarming motility of Δ*hfq* relative to SBW25 WT. **6B.** Congo Red binding of Δ*hfq*, compared to SBW25 WT and Δ*rimK*. **6C.** Glutamation assays with *E*. *coli* and SBW25 RimK, and RpsF/RpsF-D139K. The contents of each assay are indicated underneath the relevant lanes. Running positions of RimK, RpsF and glutamated-RpsF (RpsF*) are marked with arrows. Incubation time is shown above the gel image; all controls were incubated overnight. **6D.** Wheat root attachment assay for Δ*rimK*, Tn*7*::*rimK* and *rpsF-D139K*, relative to WT SBW25. **6E.** Rhizosphere colonisation competition assays for Δ*hfq*, Δ*rimK*, *rpsF-D139K* and WT SBW25. The graph shows the ratio of mutant to WT-*lacZ* CFU recovered from the rhizospheres of wheat plants seven days post-inoculation. Each dot represents CFU recovered from an individual plant. **6F.** Western blot showing RpsF levels in mutant cell lysates. Statistically significant differences between WT SBW25 and mutant strains are indicated throughout (*** = p < 0.01).

To further test whether the *P*. *fluorescens* Δ*rimK* phenotypes may be attributed to reduced Hfq abundance, an SBW25 *hfq* deletion mutant was produced and tested for phenotypes including swarming and wheat rhizosphere colonisation. Deletion of *hfq* produced small, smooth colonies that took significantly longer to arise than WT SBW25. Similarly to Δ*rimK*, the Δ*hfq* mutant showed compromised swarming motility (Fig 6A), as well as enhanced Congo Red binding (Fig 6B). Rhizosphere colonisation was also significantly compromised (Fig 6C), with too few Δ*hfq* CFUs recovered (<0.1% of WT) to quantify in some cases. The phenotypes seen upon *hfq* deletion were markedly more severe than were seen for Δ*rimK*, although this is perhaps unsurprising as Hfq is still present in the Δ*rimK* background, albeit at a reduced abundance.

Next, SBW25 WT and Δ*hfq* soluble proteomes were isolated and separated by SDS-PAGE. The region between 25 and 58 kDa (corresponding to the size range of the most strongly upregulated proteins in Δ*rimK*) was then examined by comparative MaxQuant analysis. Consistent with the results for Δ*rimK*, *hfq* deletion led to up-regulation of multiple ABC transporter subunits, stress response proteins, secretion systems and proteins involved in the production/utilization of pyoverdin and other siderophores (S3 Table). A substantial degree of overlap was seen between the Δ*rimK* and Δ*hfq* proteomes. 25 significantly upregulated proteins were common to both datasets (Table 3, S3 Table), including ten ABC transporters, two stress response proteins including SodA, and three iron homeostasis proteins. These data support the hypothesis that many of the phenotypic and proteomic changes in Δ*rimK* are ultimately the result of reduced Hfq levels in this strain.

**Table 3:** Up-regulated proteins common to both Δ*hfq* and Δ*rimK* mutants.

Up-regulated protein	Fold change in Δ*hfq*	Fold change in Δ*rimK*
*Ferripyoverdine receptor fpvA*	77.43	2.65
*Putative aminotransferase PFLU5135*	49.45	5.58
*Superoxide dismutase sodA*	11.96	3.22
*Putative exported protein PFLU3741*	7.59	4.30
*Putative ABC transport system ATP-binding protein PFLU1212*	6.51	4.25*
*Putative ABC transport system*, *substrate-binding protein PFLU2041*	6.26	2.25
*Phosphoenolpyruvate carboxykinase pckA*	6.17	2.75
*Putative uncharacterized protein PFLU6020*	5.39	2.66
*Putative sulfurylase PFLU4624*	4.85	2.53
*Putative uncharacterized protein PFLU5224*	4.83	3.42
*Dipeptide ABC transport system*, *substrate-binding protein dppA2*	4.53	6.35
*Putative iron utilization protein PFLU1089*	4.40	3.88
*Putative sugar ABC transport system*, *lipoprotein PFLU3117*	4.38	3.39
*Biopolymer transport membrane protein exbB*	3.64	6.27
*Glutamate/aspartate ABC transport system*, *periplasmic binding protein gltI*	3.31	5.30
*Putative sugar-binding exported protein PFLU3996*	3.30	3.58
*Putative uncharacterized protein PFLU5582*	3.30	4.05
*Lactoylglutathione lyase PFLU2991*	3.27	2.64
*Dipeptide ABC transport system*, *substrate-binding protein dppA3*	3.13	7.43
*Protein disulfide isomerase II PFLU5007*	3.10	3.09
*Putative oxidoreductase PFLU0041*	3.05	4.35
*Putative ABC transport system*, *exported protein PFLU0376*	2.80	3.05
*Putative hemin transport system*, *substrate-binding protein PFLU5229*	2.70	2.60
*Putative D-methionine ABC transport system*, *substrate-binding protein PFLU0068*	2.67	7.09
*Putative uncharacterized protein PFLU3219*	2.56	3.00

*denotes fold-upregulation of PFLU1213 in Δ*rimK*.

To confirm that the phenotypes associated with the *rimK* mutant arise specifically as a consequence of the loss of RpsF glutamation, we decided to abolish RpsF glutamation *in vivo* while disrupting RimK and RpsF as little as possible. To this end, we cloned and purified an allele of SBW25 RpsF with the penultimate C-terminal residue replaced with lysine (RpsF-D139K). This modification should prevent the C-terminal glutamation of RpsF [8]. We then tested the RpsF-D139K variant for glutamation by both SBW25 and *E*. *coli* RimK, and confirmed that neither protein could modify this allele *in vitro* (Fig 6C). Incidentally, RpsF-D139K consistently ran at a slightly different position to WT RpsF, which may be a consequence of neutralizing the strong negative charge of the RpsF C-terminus.

Next, we produced a chromosomal *rpsF-D139K* substitution mutant by allelic exchange, and tested it for plant association phenotypes alongside Δ*rimK*. As predicted, we observed both increased wheat root attachment and compromised rhizosphere colonisation to near-identical levels to those seen with the Δ*rimK* mutant (Fig 6D and 6E), strongly supporting the loss of *rpsF* glutamation as the major cause of the phenotypes seen in Δ*rimK*. Like Δ*rimK*, no differences in growth rate were observed between the *rpsF-D139K* strain and WT SBW25 in either rich or poor nutrient media (S5 Fig). Finally, we used a newly-raised polyclonal RpsF antiserum to examine levels of the RpsF protein in our various *rim/rpsF* mutant strains by Western blotting. Levels of the RpsF-D139K allele were comparable to WT RpsF, indicating that the non-glutamated allele is produced and stably maintained in the cell (Fig 6F), and supporting the idea that the phenotypes seen reflect the loss of modification rather than the complete loss of the RpsF protein.

## Discussion

Here we identify a new mechanism for control of protein abundance based on differential, post-translational modification of the ribosomal protein RpsF, in the plant-associated bacteria *P*. *fluorescens* and *P*. *syringae* and the human pathogen *P*. *aeruginosa*. Glutamation of RpsF by the α-l-glutamate ligase RimK induces specific, adaptive changes in the bacterial proteome through modification of ribosomal behaviour. These RimK-induced proteomic shifts play an important role in the adaptation of both commensal and pathogenic *Pseudomonas* species to environmental changes, and contribute to efficient root colonisation and plant infection. RimK catalyzes the ATP-dependent addition of glutamate residues to the C-terminus of the ribosomal protein RpsF. This modification has important impacts on the bacterial ribosome, with *rimK* deletion leading to reduced ribosomal protein levels. Surprisingly however, the loss of RimK modification does not visibly affect bacterial vitality, as growth rates and colony morphology remain unaffected in the Δ*rimK* mutant. Similarly, ribosomal RNA levels are unaffected by *rimK* deletion. Deletion of *rimK* also leads to marked downstream changes in the *P*. *fluorescens* proteome. As these proteomic shifts are not linked to corresponding changes in mRNA abundance for any of the proteins tested, we propose that RimK modification of the ribosome changes its function in such a way as to promote or suppress the translation of specific mRNAs and/or translational regulator abundance. The precise mechanism by which RimK activity affects ribosomal function, and the consequent remodeling of the proteome, is the subject of ongoing research.

While the proteomic changes that arise as a consequence of RimK activity are highly complex, many of the phenotypes and proteomic changes seen in Δ*rimK* may be confidently linked to the translational regulator Hfq, which is strongly down-regulated upon *rimK* deletion. Like Δ*rimK*, *hfq* deletion induces phenotypes including increased attachment factor production, and compromised motility and rhizosphere colonisation. Δ*hfq* also displayed significantly increased abundance of proteins associated with ABC transport, iron scavenging and utilization and oxidative stress responses, in common with the Δ*rimK* mutant. In support of this, biochemical and whole-cell analyses in various species have connected Hfq to proteomic changes associated with iron homeostasis [44], stress responses [48] and the production of multiple amino-acid ABC transporters in the context of rhizosphere adaptation. Microarray analysis of a *R*. *leguminosarum hfq* suppressor mutant shows that Hfq negatively regulates the mRNA stability of various amino-acid ABC transporters [45]. Furthermore, proteomic and transcriptomic studies in *S*. *meliloti* show Hfq repression of both solute-binding proteins and amino-acid ABC transporters [44,46]. Multiple proteins involved in amino-acid, nucleotide and carbohydrate metabolism are also up/down-regulated in the Δ*rimK* strain. Again, some of these changes may be linked to Hfq disruption. Alternatively, they may represent an adaptive response to altered amino-acid abundance, triggered by enhanced ABC transporter levels.

While reduced Hfq levels undoubtedly explain many of the Δ*rimK* phenotypes, numerous RimK-linked proteins were not identified in the published regulons for Hfq [44,46]. Altered abundance of several important regulatory proteins including RsmE, histone-like proteins and the transcriptional regulators AlgP1 and AlgP2 were seen in the Δ*rimK* mutant and may explain some aspects of Δ*rimK* behavior. While these changes may also be Hfq-mediated, it is also possible that translational regulation of some mRNAs may occur as a direct result of RimK ribosomal modification.

Critically, our data suggest that RimK modification of RpsF is not passive, but varies both with differential *rimK* transcription as the environment changes, and possibly also directly, through RimK interaction with RimA, RimB and the signalling molecule cdG. Both Rim proteins and cdG stimulate RimK_Pf_ enzyme activity *in vitro*, and thus at first glance appear to function as positive regulators of RimK. However, it is likely that the *in vivo* situation is more complex. In the case of RimB, strong activation of RimK by protein-protein interaction *in vitro* does not correspond to noticeable plant-associated phenotypes. This suggests that the relationship between these two proteins is both context-specific, and dependent on additional, as-yet undetermined factors.

RimK is further controlled by direct binding to the important signalling molecule cdG, which increases the glutamate ligase activity of the *P*. *fluorescens* protein *in vitro*. This binding seems to be widespread, with low-μM binding affinities measured for both the *P*. *fluorescens* and *E*. *coli* RimK homologs. Uniform stimulation of RimK activity by cdG binding runs counter to the generally accepted model for cdG signalling, where increased cdG levels promote sessile, persistent lifestyles over motile, virulent ones. Again, this suggests that the true relationship between cdG and RimK activation is likely to be more complex than that seen in our biochemical assays.

**Fig 7:**
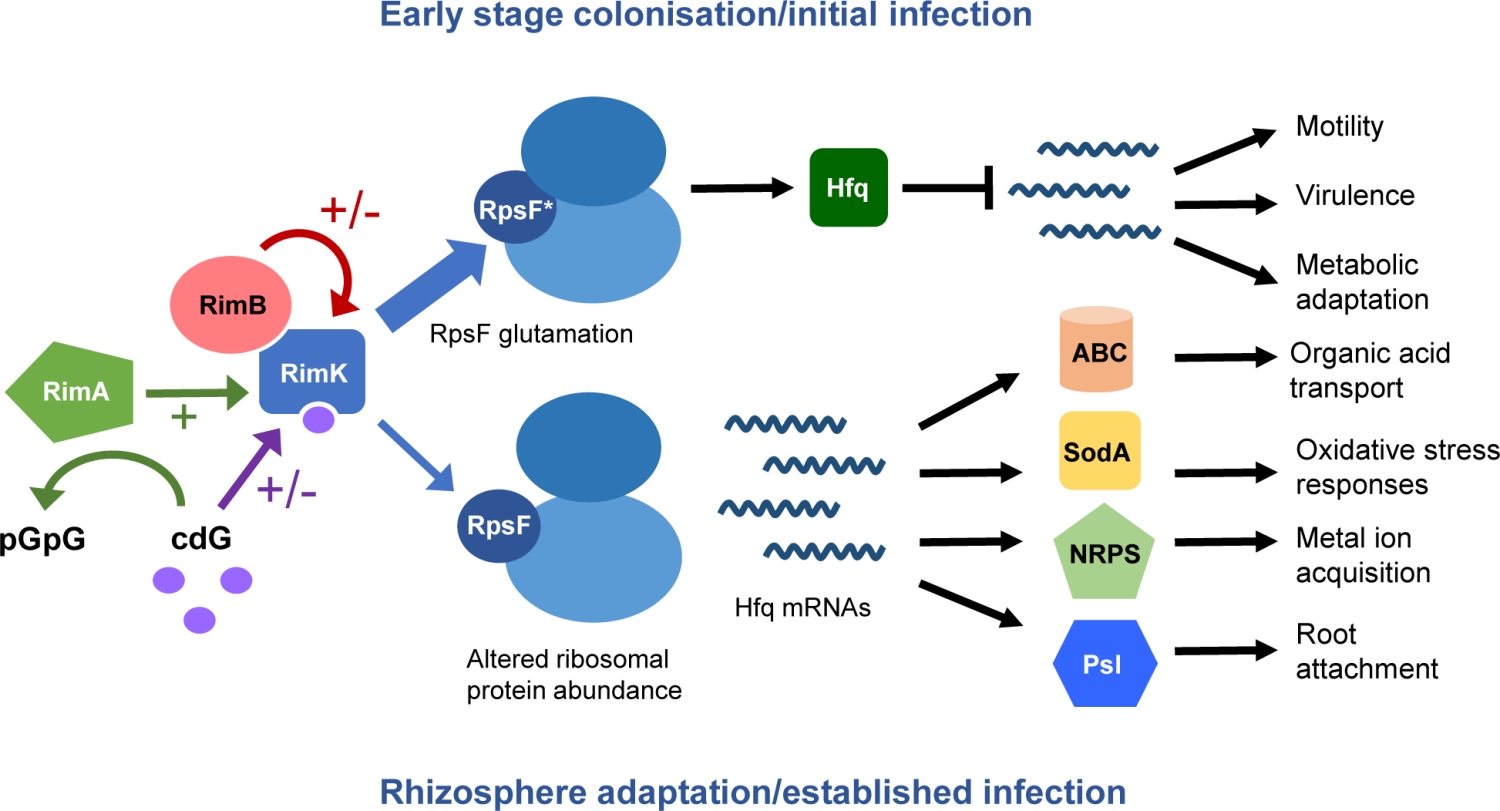
A model for RimABK function in *Pseudomonas* plant interactions. During early stage colonisation/initial infection (top row), increased RimK activity leads to increased RpsF glutamation. This leads to increased Hfq levels, with the resulting translational repression promoting phenotypes important for niche colonisation and the establishment of infection, including motility and virulence [4,44,51]. In the established rhizosphere/plant infection environment (bottom row), *rimK* transcription decreases. Less RpsF is glutamated, leading to altered ribosomal protein abundance and ribosome function, and lower Hfq levels. Release of Hfq translational repression leads to increased production of amino-acid transporters (ABC), oxidative stress response pathways (SodA), NRPSs and attachment factors. These changes promote resource acquisition, stress resistance and root attachment, and prioritize long-term adaptation to the plant environment. RimK activity is further regulated by direct interaction with the RimA/B proteins and the signalling molecule cdG. ‘+/-‘ denotes cases where the nature of protein/dinucleotide control is currently undefined.

Deletion of *rimA* produces effects consistent with a loss of *rimK in vivo*, suggesting that RimA functions as a positive regulator of RimK. While *rimB* is associated with *rimK* in around half of the genomes encoding a Rim system, *rimA* is restricted to plant-associated *Pseudomonas* species such as *P*. *fluorescens* and *P*. *syringae* [56]. For example, the *P*. *aeruginosa rim* operon lacks a *rimA* homolog. RimA thus appears to represent a particular refinement of the regulatory system required for colonisation of the complex plant-associated niche. The relationship between RimK and RimA is probably based on more than just the modest, direct stimulation of RimK activity seen *in vitro*. It seems likely that RimA moderates the relationship between cdG and RimK in some way, although the nature of this control is currently unclear. RimA may play an active role; hydrolyzing RimK-associated cdG under certain circumstances, or a passive one; constitutively degrading cdG to buffer the impact of changing dinucleotide levels on RimK activity. Alternatively, it may act as a trigger enzyme, with cdG hydrolysis altering the RimA-RimK relationship in common with a recently characterized pathway in *E*. *coli* [57]. While the details of the RimABK-cdG regulatory circuit remain to be fully established, it is clear that control of RimK activity represents a high-level regulatory mechanism that integrates cdG-signalling with post-translational ribosome modification, and hence control of bacterial protein production. In turn, this enables bacteria to rapidly fine tune their proteomes to optimally respond to the surrounding environment (Fig 7).

In our experiments, *P*. *fluorescens rimK* mRNA abundance peaked during initial wheat rhizosphere colonisation, but then progressively declined as the rhizosphere community matured over the next six days. We propose that enhanced RimK levels during the initial stages of root colonisation equates to enhanced RpsF glutamation. This in turn leads to increased abundance of Hfq (and possibly other regulators such as RsmE), and reduced translation of target mRNAs including those for ABC transporters and NRPS pathways. Our model suggests that an early peak in *rimK* expression promotes a state where organic acid transport, siderophore utilization and a sessile, surface-adherent lifestyle are downplayed in favor of increased motility and rapid colonisation of the spatial environment of the rhizosphere (Fig 7). Reduced *rimK* transcription in the established wheat rhizosphere would lead to decreased Hfq abundance, and consequently to increased root attachment, production of siderophores and deployment of ABC transporters for polyamines, peptides and organic acids (Fig 7). Organic acid catabolism is the predominant form of carbon metabolism in the rhizosphere [17], necessitating a shift towards ABC transporter expression and metabolic enzyme reorganization. Similarly, siderophore production plays an important survival role in the competitive rhizosphere environment [14], scavenging iron and other metals and providing a measure of oxidative stress-protection [58]. Reduced RimK/Hfq abundance in the established rhizosphere also corresponds to up-regulation of oxidative stress response proteins such as SodA. Again, this is logical in the context of a global strategy for adaptation to the rhizosphere environment, where oxidative stress is a constant threat [59]. Induction of the *soxR* regulon has been shown upon RpsF glutamation in *E*. *coli*, and a similar mechanism may function in *P*. *fluorescens* [60]. Down-regulating RimK production may therefore represent an effective strategy for optimizing the bacterial proteome to the established rhizosphere environment (Fig 7).

RimABK regulatory effects appear to be both subtle and global; ‘tuning’ the ribosome for optimal performance in a particular environment rather than acting as a checkpoint for a specific phenotype in response to an input signal. This is reflected in the relatively modest phenotypes seen for *P*. *fluorescens* Δ*rimK* compared to Δ*hfq*. For example, compromised rhizosphere colonisation by Δ*rimK* is likely to stem from multiple interconnected factors, including an inability to fully adapt the surface transporter complement to optimally exploit the organic-acid rich environment of the rhizosphere [17], putrescine transporter overproduction leading to H_2_O_2_ toxicity [61,62], and disruption of the complex relationship between motility and attachment during rhizoplane colonisation [6,14,63].

In addition to controlling phenotypes associated with colonisation and metabolic adaptation, RimK plays an important role in the virulence of both human and plant pathogenic pseudomonads. In *P*. *syringae*, RimK is important for the initial stages of plant infection but is not required for apoplastic proliferation. This is in agreement with the results seen for *rimK* expression during *P*. *fluorescens* rhizosphere colonisation, and suggests that RimK fulfils a similar, global adaptation role in both species. Thus, RimK activity in the early stages of infection would promote bacterial migration from plant surfaces into the apoplast through stomata and wounds on the plant surface. Once infection is underway, *P*. *syringae* changes the expression of metabolic genes to exploit the nutrient availability of the apoplast [64,65], and up-regulates multiple additional loci including genes for polysaccharide synthesis, stress tolerance and nutrient uptake [65]. RimK would therefore become redundant under these conditions. For the opportunistic human pathogen *P*. *aeruginosa*, the loss of virulence associated with *rimK* deletion was more general; occurring even in stabbed lettuce leaves and accompanied by the loss of β-hemolytic activity. This suggests that virulence loci are directly under *rimK*/*hfq* control in *P*. *aeruginosa*. While significant parallels exist between the RimK regulons of *P*. *fluorescens*, *P*. *syringae* and *P*. *aeruginosa*, there are nonetheless also important differences between them. We are actively investigating the differences between the Δ*rimK* proteomes of various *Pseudomonas* species, and how they impact on the relationship between pathogenic and commensal microbes and their respective hosts.

## Methods

### Strains and growth conditions

Strains and plasmids are listed in S4 Table. Primers are listed in S5 Table. Unless otherwise stated all *P*. *fluorescens* and *P*. *syringae* strains were grown at 28°C, and *P*. *aeruginosa* and *E*. *coli* at 37°C in lysogenic broth (LB) [66], solidified with 1.3% agar where appropriate. Gentamycin was used at 25 μg/ml, carbenicillin at 100 μg/ml, piperacillin and fosfomycin at 2 mg/ml and tetracycline (Tet) at 12.5 μg/ml (50 μg/ml for *P*. *aeruginosa*). For inducible plasmids, IPTG was added to a final concentration 0.2 (SBW25) or 1 mM (*E*. *coli*) as appropriate.

### Molecular biology procedures

Cloning was carried out in accordance with standard molecular biology techniques. The pME-*rimA/B/K* plasmids were constructed by ligation of the appropriate PCR fragments (amplified with primers 5/6, 3/4 and 1/2 respectively, from SBW25 genomic DNA), between the *Eco*RI and *Kpn*I sites of plasmid pME6032 [67]. The C-terminal flag-tagged *rim* plasmids were produced from these by the method described by Yu et al. following flag cassette amplification from pSUB11 with primers 9/10, 8/10 and 7/10 [68]. The kanamycin gene inserted downstream of the *rim* gene was then excised by transformation of the *E*. *coli* host with pFLP2 [69] followed by sucrose counter-selection. The pETM11-*rpsF*, pETM11-*rimB*, pETM11-*rimA* and pET42b(+)-*rimK* purification vectors were produced by ligating PCR fragments (amplified with primers 21/22, 29/30, 13/14, 15/16, 11/12 and 27/28) between the *Nde*I and *Xho*I sites of plasmids pET*Nde*M-11 [70] and pET42b(+) (Novagen) as appropriate. Reverse-transcriptase PCR (RT-PCR) was conducted using primers (31–52). The SBW25 *rimABK* complementation vectors were constructed by ligation of the relevant *rimABK* PCR fragments (amplified from appropriate *rim* deletion strains with primers 74–77) between the *Hin*dIII and *Bam*HI sites of pUC18T-mini-Tn*7*T-Gm.

### Gene deletions

*P*. *fluorescens*, *P*. *syringae* and *P*. *aeruginosa* deletion mutants were constructed via an adaptation of the protocol described elsewhere [71]. Up- and downstream flanking regions to the target genes were amplified using primers 17–20, 53–56, 57–60, 23–26 and 61–64. PCR products in each case were ligated into pME3087 between *Eco*RI-*Bam*HI. The resulting vectors were transformed into the target strain, and single crossovers were selected on Tet and re-streaked. Cultures from single crossovers were grown overnight in LB medium, then diluted 1:100 into fresh medium. After 2 hours, 5 μg/ml Tet (20 μg/ml for *P*. *aeruginosa*) was added to inhibit the growth of cells that had lost the Tet cassette. After a further hour of growth, samples were pelleted and re-suspended in fresh LB containing Tet and 2 mg/ml piperacillin and phosphomycin to kill growing bacteria. Cultures were grown for a further 4–6 hours, washed once in LB and a dilution series plated onto LB agar. Individual colonies were patched onto LB plates ± Tet, and Tet-sensitive colonies tested for gene deletion/modification by colony PCR.

### *rpsF-D139K* point mutation

The mutant allele with flanking regions for the SBW25 *D139K* point mutation in *rpsF* (*PFLU0533*) was prepared by primer extension PCR. Primer pairs 66/68 and 67/69 were used to produce PCR products that were subsequently combined in a second PCR with primer pair 66/69 to produce the mutant allele with flanking regions. This product was ligated into pME3087 between *Xho*I-*Bam*HI and introduced into the *P*. *fluorescens* chromosome as detailed above.

### Phenotypic assays

The Congo Red (CR) binding assay was adapted from [72]. Five 10 μl drops of LB overnight cultures per strain were grown on 20 ml King’s B agar plates for 24 hours at 28°C. Each colony was then re-suspended in 1 ml 0.005% (w/v) CR (Sigma) and incubated for 2 h at 37°C with shaking. Colony material was pelleted by centrifugation and CR remaining in the supernatant determined by measurement of A_490_ compared to appropriate CR standards. To measure swarming motility, 0.3–0.5% Kings B agar plates (as indicated) were poured and allowed to set and dry for 1 hour in a sterile flow chamber. Plates were then inoculated with 2 μl spots of overnight cultures, and incubated overnight at room temperature. Each sample was tested in triplicate. Disc inhibition assays were carried out using paper discs impregnated with 20 μg/ml gentamycin, on LB + 0.004% CR plates spread with 100 μl of OD 1.0 overnight cultures. Plates were then incubated overnight at 28°C. Assays were repeated at least once independently, and statistical significance assessed using Students t-tests where appropriate.

### Growth assays

Bacterial growth was monitored in a microplate spectrophotometer (BioTek Instruments) using a minimum of 3 experimental replicates/sample. Wells (of a 96-well plate) contained 150μL of the indicated growth medium, supplemented with 0.1 mM IPTG and 12.5 μg/ml tetracycline where appropriate. For the antibiotic inhibition assays (S3 Fig), gentamycin, kanamycin and carbenicillin were added to the concentrations noted. Growth was initiated by the addition of 5μL of cell culture with an OD_600_ = 0.01. Plates were covered with adhesive sealing sheets and incubated statically at 28°C.

### Wheat root attachment

SBW25 strains containing the bioluminescent plasmid pIJ-11-282 [73] were grown overnight in M9 0.4% pyruvate media. Cultures were normalized by luminescence using a GloMax Multi JR luminometer (Promega) and diluted 1:100 in 10 ml 25 mM pH7.5 phosphate buffer in sterile 50 ml tubes, each containing 12–15 1.5 cm long sterile 3 day-old wheat root tips. Tubes were incubated for 2 hours at room temperature with gentle shaking, before supernatant was discarded and the roots washed twice with phosphate buffer. 10 roots per sample were transferred to individual tubes and luminescence measured and compared with that obtained for wild-type SBW25. The assay was repeated twice independently, and statistical significance assessed using Students t-tests.

### Rhizosphere colonization

Paragon wheat seeds were sterilized with 70% ethanol and 5% hypochlorite, washed, and germinated on sterile 0.8% MS agar for 72 h in the dark. Seedlings were then transferred into sterile 50 ml tubes containing medium grain vermiculite and rooting solution (1 mM CaCl_2_.2H_2_O, 100 μM KCl, 800 μM MgSO_4_, 10 μM FeEDTA, 35 μM H_3_BO_3_, 9 μM MnCl_2_.4H_2_O, 0.8 μM ZnCl_2_, 0.5 μM Na_2_MoO_4_.2H_2_O, 0.3 μM CuSO_4_.5H_2_O, 6 mM KNO_3_, 18.4 mM KH_2_PO_4_, and 20 mM Na_2_HPO_4_), and transferred to a controlled environment room (25°C, 16 h light cycle). WT-*lacZ* and mutant SBW25 strains were grown overnight in M9 0.4% pyruvate media, then diluted in phosphate buffer and 1 x 10^3^ CFU of mutant and WT-*lacZ* bacteria used to inoculate seven day-old seedlings. Plants were grown for a further seven days, after which shoots were removed, 20 ml PBS was added to each tube and vortexed thoroughly to resuspend bacteria. A dilution series was plated onto XGal + IPTG plates and WT-*lacZ*/mutant colonies distinguished by blue/white selection. Assays were conducted for 8–12 plants/mutant, repeated at least twice independently, and statistical significance assessed using Mann-Whitney tests.

### Bacterial plant infection

*P*. *aeruginosa* lettuce leaf infections were carried out after [36]. For *P*. *syringae* infections, *Arabidopsis thaliana* ecotype *Columbia* (Col-0) plants were grown at 20–22°C under 10 h light period for 4 weeks. *Pto* DC3000 cultures were grown overnight, re-suspended in 10 mM MgCl_2_ and adjusted to OD_600_ = 0.0002 (10^5^ CFU/ml) for syringe infiltration and OD_600_ = 0.05 (10^7^ CFU/ml) for spray infection. Shortly before spraying 0.02% Silwet L77 was added to the suspension, plants were sprayed until run-off. All plants were covered with vented lids for five days. Six leaf discs (7 mm diameter) from six different plants per strain were collected in 10 mM MgCl_2_ and homogenized using a drill-adapted pestle. Serial dilutions were plated and CFUs determined in each case. The assay was repeated three times independently and statistical significance assessed using Students t-tests.

### Co-immunoprecipitation

SBW25 pME-*rimA/B/K-flag* overnight cultures were pelleted by centrifugation, re-suspended in ice-cold IP buffer (20 mM HEPES pH 7.4, 100 mM NaCl, 1 mM EDTA, 1.0% ^v^/_v_ Triton X-100, protease inhibitor), and incubated at 4°C with end-over-end agitation for 6 hours. Samples were then centrifuged (15,000 g, 20 min, 4°C), and the supernatant removed and incubated with 20 μg/ml protein-A agarose beads (4°C, end-over-end agitation, 30 min) to remove non-specifically binding proteins. Samples were then centrifuged (3,000 g, 1 min 4°C) to pellet the beads, an aliquot of the supernatant was taken for analysis, and the remaining supernatant was incubated overnight with 20 μg/ml ANTI-FLAG M2 affinity gel (Sigma) (4°C, end-over-end agitation). Samples were pelleted by centrifugation (3,000 g, 1 min 4°C), the supernatant was discarded and the beads re-suspended in 1.0 ml ice-cold IP buffer. This wash step was repeated 5 times. The beads were then re-suspended in SDS sample buffer, boiled, and pelleted by centrifugation. The presence of Flag-tagged Rim proteins in the supernatant was confirmed by immunoblotting, and interacting proteins were detected by Orbitrap mass spectrometry. Results were compared with two control datasets (M2 bead-only, and a non-specific protein control based on immunoprecipitation of unrelated flag-tagged proteins (PFLU3129 and PFLU3130)).

### Ribosomal enrichment

Ribosomal enrichment was adapted from the method described in [74]. 500 ml SBW25 WT and Δ*rimK* overnight cultures were grown in M9 0.4% pyruvate. 1 mM chloramphenicol was added, and samples were incubated on ice for 20 minutes. Cells were pelleted by centrifugation, re-suspended in 2ml lysis buffer (20 mM Hepes pH 7.8, 6 mM MgCI_2_, 100 mM NaCI, 16% (^w^/_v_) sucrose), and incubated on ice with 2 mg/ml lysozyme for 1 hour. Samples were then sonicated for 30 seconds, and centrifuged twice (10,000 g, 15 min, 4°C) to remove cell debris. The resulting lysates were diluted threefold with running buffer (20 mM Hepes pH 7.8, 6 mM MgCI_2_, 100 mM NaCI, 2 mM EDTA, protease inhibitor), loaded onto 2 ml 35% sucrose cushions and ultracentrifuged for 2 hours (300,000 g, 4°C). Pellets from the ultracentrifugation step were washed twice and re-suspended in running buffer before analysis by SDS-PAGE and Orbitrap mass spectrometry.

### Purification of Rim and RpsF proteins

1.0 litre *E*. *coli* BL21-(DE3) pLysS overexpression cultures were inoculated from overnights and grown at 30°C to an OD_600_ of 0.6, before protein expression was induced for 2 hours with 1mM IPTG. Cells were then lysed by French press and His_6_-tagged proteins purified by NTA-Ni chromatography. 1 ml HiTrap chelating HP columns (Amersham) were equilibrated with 25 mM KH_2_PO_4_, 200 mM NaCl, pH 8.0 (SBW25 RimA/B/K), 50 mM Tris-Cl, 2.5% glycerol, pH 8.0 (SBW25 RpsF) or 50 mM Tris, 300 mM NaCl, 10 mM imidazole, pH 9.0 (*E*. *coli* RimK and RpsF), and loaded with cell lysate. Following protein immobilization, elution was accomplished using a linear gradient to 500 mM imidazole over a 15 ml elution volume.

### RpsF glutamation assays

The glutamation assay was adapted from Kino et al. [9]. Briefly, purified RpsF and Rim proteins in a 1:1 ratio were incubated for the indicated times at room temperature in reaction buffer (20 mM glutamate, 20 mM ATP, 20 mM MgSO_4_·7H_2_O, 100 mM Tris-HCl pH 9). The reaction products were then analyzed using Tricine-SDS-PAGE gels and MALDI-TOF spectroscopy. Reactions were supplemented as indicated with a 1:1 ratio of purified RimA/RimB, 150 μM cdG (Sigma), and/or 13 μM L-[^14^C(U)]-Glutamic Acid (Perkin Elmer). For the experiments in Fig 4F, assays were repeated in triplicate and radiolabel incorporation was quantified using MultiGauge software (Fuji Film).

### Linked Pyruvate Kinase / Lactate Dehydrogenase (PK/LDH) ATPase activity assay

ATPase activity was measured indirectly by monitoring NADH oxidation. The reaction buffer consisted of 50 mM Tris-Cl (pH 8.0), 2 mM MgCl2, 1 mM DTT and 10mM KCl. Each 100 μL reaction contained 0.4 mM NADH, 0.8 mM phosphoenolpyruvic acid, 1 μM RimK/RpsF protein, 0.7 μl PK/LDH (Sigma) and was initiated by the addition of 10 μL ATP. Enzyme kinetics were determined by measuring A_340_ at 1 minute intervals. Kinetic parameters were calculated by plotting the specific activity of the enzyme (nmol ATP hydrolysed/ min/ mg of protein) versus ATP concentration and by fitting the non-linear enzyme kinetics model (Hill equation) in GraphPad Prism. 25 μM cdG or 1 μM RimB/RimA proteins were included as appropriate.

### Phosphodiesterase activity assay

The assay was carried out after Christen et al. [75]. Briefly, ^32^P-cdG was produced enzymatically with PleD*, then 100 μM cdG supplemented with 0.07 μM ^32^P-cdG was incubated with 1.5 μM YhjH, RimA or BSA at 30°C. The reaction buffer contained 25 mM Tris pH 8.0, 100 mM NaCl, 10 mM MgCl_2_, 5 mM β-mercaptoethanol and 5% glycerol. Aliquots were removed after 5 and 60 min, and the reaction stopped with 0.25 M EDTA before TLC separation and visualization.

### Biotinylated cdG pull-down experiment

Cell lysates overexpressing RimK were prepared by sonicating 5 ml of culture, previously induced with 0.5mM IPTG for 5 hours at 28°C. The lysed cells were pelleted (20,000 g, 1 hr.) and 45 μL of the soluble fraction was collected and mixed with biotinylated cdG (BioLog B098) at a final concentration of 30 μM. Samples were then incubated overnight with end-over-end rotation at 8°C. Samples were then cross linked for 4 minutes in a UV Stratalinker (Stratagene) before addition of 25μl of streptavidin magnetic beads (Invitrogen) and a further hour of incubation with end-over-end rotation at 8°C. Streptavidin magnetic beads were recovered with a magnet and washed five times with 200 μL of protein wash buffer (20 mM HEPES pH 7.5, 250 mM NaCl, 2 mM MgCl_2_, 2.5% (v/v) glycerol), to remove unbound proteins. Samples were then run on an SDS-PAGE gel and visualized with colloidal Coomassie stain.

### Surface plasmon resonance

SPR experiments were conducted at 25°C with a Biacore T200 system using a Streptavidin SA sensor chip (GE healthcare) with four flow cells each containing SA pre-immobilized to a carboxymethylated dextran matrix. Flow cell one (FC1) and flow cell three (FC3) were kept blank for reference subtraction. The chip was first washed three times with 1 M NaCl, 50 mM NaOH to remove unconjugated streptavidin. 100 nM biotinylated cdG (BioLog B098) was then immobilised on FC2 and FC4 at a 50 RU immobilisation level with a flow rate of 5 μL/min. Soluble RimK alleles at the required concentration were prepared in SPR buffer (10 mM HEPES, 150 mM NaCl, 0.1% Tween 20, 2 mM MgCl_2_, pH 6.5). Samples were injected with a flow rate of 5 μL/min over the reference and cdG cells for 90 seconds, followed by buffer flow for 60 seconds. The chip was washed at the end of each cycle with 1 M NaCl. Replicates for each protein concentration were included as appropriate. Sensorgrams were analysed using Biacore T200 BiaEvaluation software 1.0 (GE Healthcare).

### Quantitative analysis using isobaric labelling (iTRAQ)

50 ml SBW25 WT and Δ*rimK* overnight cultures were grown in M9 0.4% pyruvate. Cellular activity was then frozen by addition of 30 ml of ‘RNAlater’ (saturated (NH_4_)_2_SO_4_, 16.7 mM Na-Citrate, 13.3 mM EDTA, pH 5.2) and protease inhibitors. Cells were pelleted by centrifugation and washed three times with 10 mM HEPES pH 8.0 + protease inhibitors, before re-suspension in 200 μL. 700 μL pre-cooled RLT + β-mercaptoethanol buffer (RNeasy Mini Kit, QIAGEN) was added and samples lysed with two 30 second Ribolyser ‘pulses’ at speed 6.5. The supernatant was removed, and the soluble fraction separated by ultracentrifugation (279,000 g, 30 minutes, 4°C). Soluble proteomes were acetone precipitated and protein concentrations determined. The proteomic samples were then subjected to iTRAQ quantitative mass spectrometry.

Specifically, samples were reduced, alkylated and digested with trypsin [76], then labelled with iTRAQ tags according to the manufacturer’s instructions (AB Sciex). Samples were then mixed, desalted on a SepPak column (Waters) and fractionated by high-pH reversed phase chromatography on an Xterra HPLC column (Waters). The fractionated samples were analyzed by LC-MS/MS on a Synapt G2 mass spectrometer coupled to a nanoAcquity UPLC system (Waters). Peaklist (.pkl) files were generated in ProteinLynx Global Server 2.5.2 (Waters).

### Label-free analysis of protein extracts by LC-MS/MS

Ribosome protein samples were acetone precipitated and re-dissolved in 8 M urea, 100 mM Tris-HCl pH 8.0. Eluates from Co-IPs were run into an SDS gel and bands cut out for protein identification. All samples were reduced, alkylated, and digested with trypsin [76], then analyzed by LC-MS/MS on an LTQ-Orbitrap™ mass spectrometer (Thermo Fisher) coupled to a nanoAcquity UPLC system (Waters). Data dependent analysis was carried out in Orbitrap-IT parallel mode using CID fragmentation of the 5 most abundant ions in each cycle. The Orbitrap was run with a resolution of 30,000 over the MS range from m/z 350 to m/z 1800, an MS target of 10^6^ and 1 s maximum scan time. The MS2 was triggered by a minimal signal of 1000 with an AGC target of 3x10^4^ ions and 150 ms scan time using the chromatography function for peak apex detection. Dynamic exclusion was set to 1 count and 60 s exclusion with an exclusion mass window of ±20 ppm. Raw files were processed with MaxQuant version 1.5.0.30 (http://maxquant.org). For relative label-free quantitation (LFQ) the following parameters were used: min. unique peptides = 2, peptides for quantitation = unique, include oxidised (M) peptides, maximum missed cleavages = 1, min. LFQ ratio count = 1, match between runs = yes, intensity determination = sum FWHM (smooth).

### Protein identification from gel bands by MALDI-TOF

Gel slices were treated and digested with trypsin [76] and peptides spotted onto a PAC II plate (Bruker Daltonics). The spots were washed briefly with 10 mM NH_4_PO_4_, 0.1% TFA according to the manufacturer, dried and analyzed by MALDI-TOF on an Ultraflex TOF/TOF (Bruker). The instrument was calibrated using the pre-spotted standards (ca. 200 laser shots). Samples were analyzed using a laser power of approx. 25%, and spectra were summed from ca. 10 x 30 laser shots. Data processing was conducted using FlexAnalysis (Bruker).

Database searches in each case were performed using an in-house Mascot 2.4 Server (Matrixscience) on a *P*. *fluorescens* protein database (www.uniprot.org). Mascot search results were imported into Scaffold (Proteome Software) for evaluation and comparison. Mass spectrometry data have been deposited to the ProteomeXchange Consortium (http://proteomecentral.proteomexchange.org) via the PRIDE partner repository [77] [Dataset identifiers PXD001371 and PXD001376, Project DOI: 10.6019/PXD001376, PXD002573, Project DOI: 10.6019/PXD002573].

### RNA extraction

To obtain SBW25 rhizosphere RNA, shoots were removed from 8 wheat seedlings, each previously inoculated with 10^8^ CFU bacteria. 20 ml of 60% RNAlater (in PBS) was added to each tube, and sealed tubes were vortexed for 10 min at 4°C. The 8 samples were combined and filtered through 4 layers of muslin into sterile tubes, and the filtrate was centrifuged (200 g, 4°C, 1 min) to remove heavy particulate contamination. Cells were then pelleted (10,000 rpm, 4°C, 10 min) and lysed by mechanical disruption before RNA was purified from the lysate by column capture (QIAGEN RNeasy Mini Kit). Purified RNA was subjected to an additional DNase treatment (Turbo™ DNase, Ambion). RNA quantification was performed by specific fluorometric quantitation (Qubit®, Life Technologies).

### Quantitative real-time PCR (qRT-PCR) analysis

cDNA synthesis was performed using SuperScript II reverse transcriptase and random primers (Invitrogen) in the presence of RNasin ribonuclease inhibitor (Promega). The quantity of total RNA used was dictated by the lowest concentration sample in each assay in the case of rhizosphere samples. cDNA was then used as template in qRT-PCR performed with a SensiFAST SYBR No-ROX kit (Bioline). Three technical replicates were used for each gene. Specific qPCR primers (31–34 and 70–73) were used to amplify reference and target genes. To normalize for differing primer efficiency, a standard curve was constructed (in duplicate) using chromosomal DNA. Melting curve analysis was used to confirm the production of a specific single product from each primer pair. qRT-PCR was performed using a CFX96 Touch instrument using hard-shell white PCR plates (Bio Rad). PCR products were detected with SYBR green fluorescent dye and amplified according to the following protocol: 95°C, 3 min, then 50 cycles at 95°C 5 sec, 62°C 10 sec and 72°C 7 sec. Melting curves were generated at 65 to 95°C with 0.5°C increments. Primers were used at a final concentration of 1 μM. The entire experiment (including RNA extraction) was repeated once independently.

## Supporting Information

S1 Fig mRNA abundance and growth curves for Δ*rimA*, *B* and *K* mutants. **A.** *rimK* mRNA abundance in Δ*rimA*, *B* and *K* mutant backgrounds, relative to WT SBW25. **B.** Growth curves for SBW25 WT and Δ*rimA*, *B* and *K* in rooting solution + 0.4% pyruvate, 0.4% glucose (see Methods, Rhizosphere colonisation). **C.** Growth curves for SBW25 WT and Δ*rimA*, *B* and *K* in rooting solution + 0.4% pyruvate. **D.** Growth curve for PA01 and Δ*rimK* in rooting solution + 0.4% pyruvate, 0.4% glucose. No significant differences in growth rate were seen between WT and any of the *rim* mutants under the tested conditions. Experiments were repeated at least twice independently.
(TIF)

S2 Fig ATPase activity of *E*. *coli* His_6_-RimK (RimK_Ec_) incubated with glutamate. RimK_Ec_ specific activity (nmol ATP hydrolyzed/min/mg RimK) is shown for increasing concentrations of ATP.
(TIF)

S3 Fig *rimK* overexpression effects on antibiotic susceptibility. **A.** Growth curves in rooting solution + 0.4% pyruvate (RSP) plus gentamycin (Gent) for SBW25 either containing an empty vector (WT) or overexpressing *rimK* (p-*rimK*). **B.** Growth curves in RSP plus kanamycin (Kan) for SBW25 either containing an empty vector (WT) or overexpressing *rimK* (p-*rimK*). **C.** Growth curves in RSP plus phosphomycin (Phos) for SBW25 either containing an empty vector (WT) or overexpressing *rimK* (p-*rimK*). **D.** Gentamycin disc inhibition assays for SBW25 containing empty vector (WT) or overexpressing *rimK* (p-*rimK*). Congo Red dye was added to the plates to improve contrast.
(TIF)

S4 Fig CdG effects on RimK. **A.** SPR sensorgram and affinity data for RimK_Ec_ binding to biotinylated cdG. A range of RimK_Ec_ concentrations was used (1.25, 2.5, 5, 10, 20, and 40 μM) and concentration replicates included as appropriate together with buffer only controls. Protein binding and dissociation phases are shown. For the affinity fit, binding responses were measured 4s before the end of the injection and *K*_*d*_ values for each protein calculated using BiaEvaluation software, and confirmed using GraphPad. **B.** ATPase activity of RimK_Pf_ incubated with glutamate and cdG. RimK_Pf_ specific activity (*V*_*max*_ = 234.9 nmol ATP/min/mg protein) is shown for increasing concentrations of ATP (squares). Addition of 25 μM cdG (circles) increases *V*_*max*_ to 471.0 nmol ATP/min/mg.
(TIF)

S5 Fig The *rpsF-D139K* mutation does not affect growth. Growth curves for SBW25 WT, Δ*rimK* and *rpsF-D139K* in rooting solution + 0.4% pyruvate, ± 0.4% glucose. No significant differences in growth rate were seen between WT and *rpsF-D139K* in either condition.
(TIF)

S1 Table Ribosomal proteins detected in SBW25 WT and Δ*rimK*. (DOCX)

S2 Table Up and down-regulated proteins in SBW25 Δ*rimK*. (DOCX)

S3 Table Up-regulated proteins in SBW25 Δ*hfq*. (DOCX)

S4 Table Strains and plasmids. (DOCX)

S5 Table Primers. (DOCX)

S6 Table Relative protein abundances in SBW25 WT and Δ*rimK*. (XLSX)
