# Supplementary figures and images for: Adaptive Remodeling of the Bacterial Proteome by Specific Ribosomal Modification Regulates Pseudomonas Infection and Niche Colonisation
Source: PLoS Genet. 2016 Feb 4;12(2):e1005837. doi: 10.1371/journal.pgen.1005837 (PMC4741518; doi:10.1371/journal.pgen.1005837)

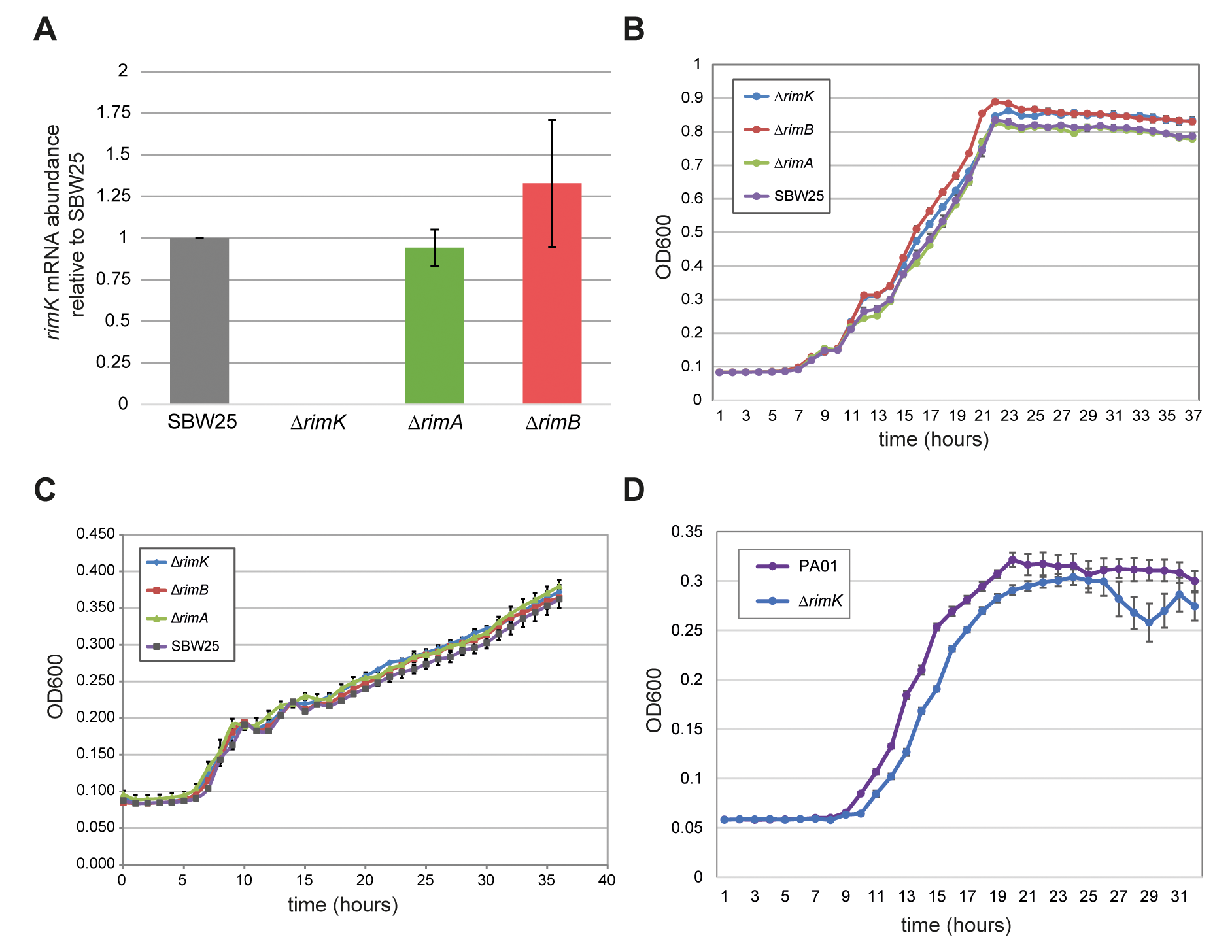

Supplement: S1 Fig — A. rimK mRNA abundance in ΔrimA, B and K mutant backgrounds, relative to WT SBW25. B. Growth curves for SBW25 WT and ΔrimA, B and K in rooting solution + 0.4% pyruvate, 0.4% glucose (see Methods, Rhizosphere colonisation). C. Growth curves for SBW25 WT and ΔrimA, B and K in rooting solution + 0.4% pyruvate. D. Growth curve for PA01 and ΔrimK in rooting solution + 0.4% pyruvate, 0.4% glucose. No significant differences in growth rate were seen between WT and any of the rim mutants under the tested conditions. Experiments were repeated at least twice independently. (TIF) [file pgen.1005837.s001.tif]

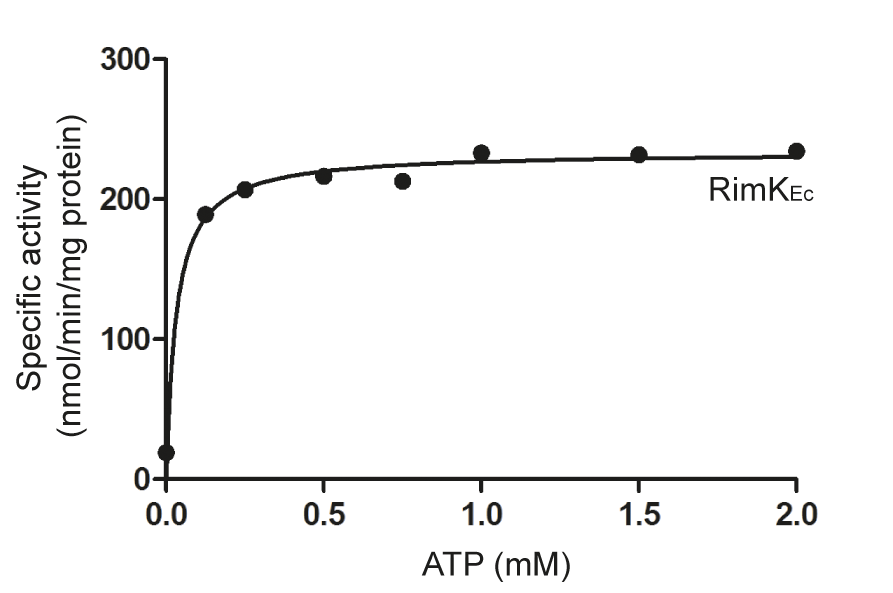

Supplement: S2 Fig — RimKEc specific activity (nmol ATP hydrolyzed/min/mg RimK) is shown for increasing concentrations of ATP. (TIF) [file pgen.1005837.s002.tif]

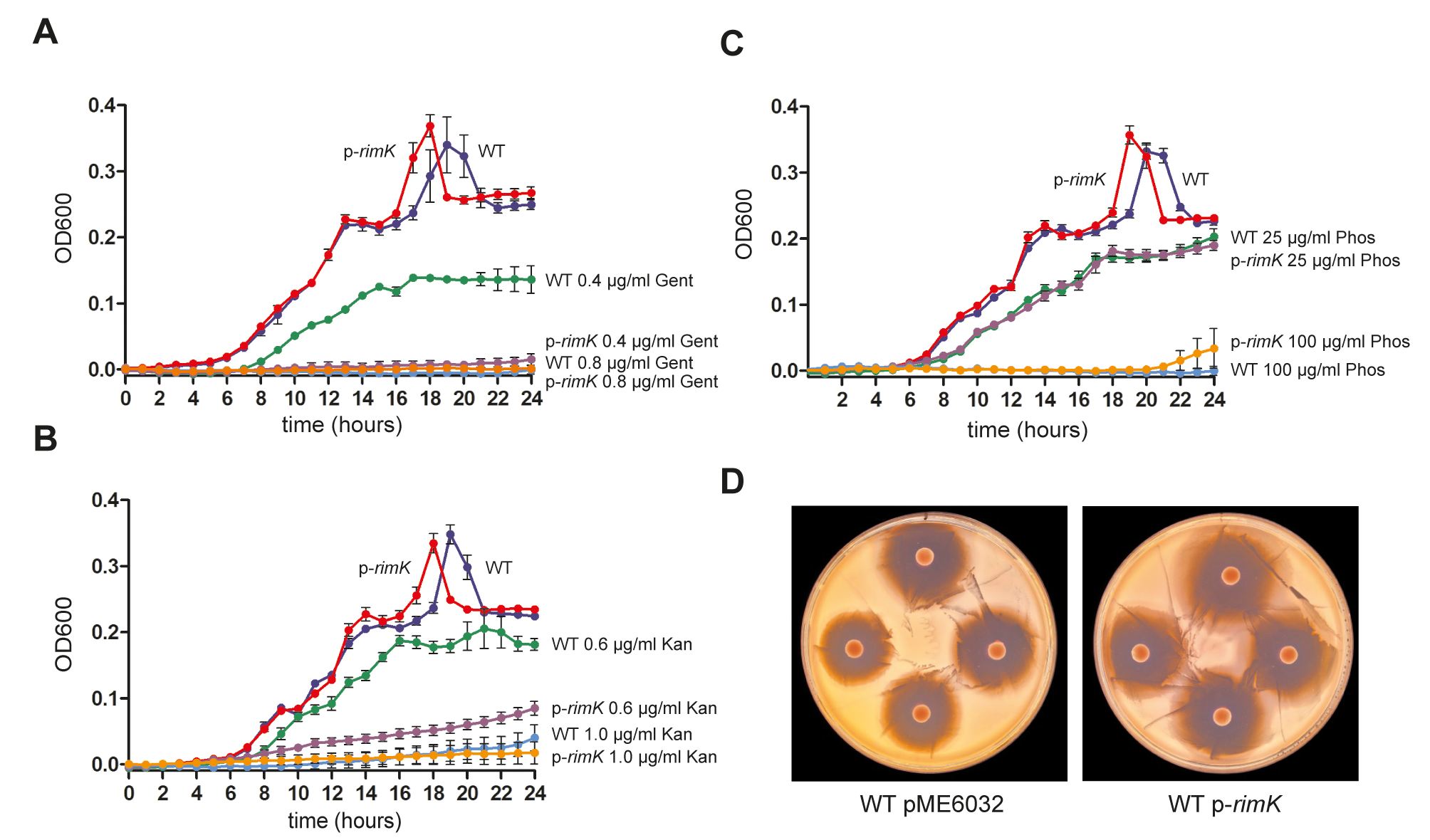

Supplement: S3 Fig — A. Growth curves in rooting solution + 0.4% pyruvate (RSP) plus gentamycin (Gent) for SBW25 either containing an empty vector (WT) or overexpressing rimK (p-rimK). B. Growth curves in RSP plus kanamycin (Kan) for SBW25 either containing an empty vector (WT) or overexpressing rimK (p-rimK). C. Growth curves in RSP plus phosphomycin (Phos) for SBW25 either containing an empty vector (WT) or overexpressing rimK (p-rimK). D. Gentamycin disc inhibition assays for SBW25 containing empty vector (WT) or overexpressing rimK (p-rimK). Congo Red dye was added to the plates to improve contrast. (TIF) [file pgen.1005837.s003.tif]

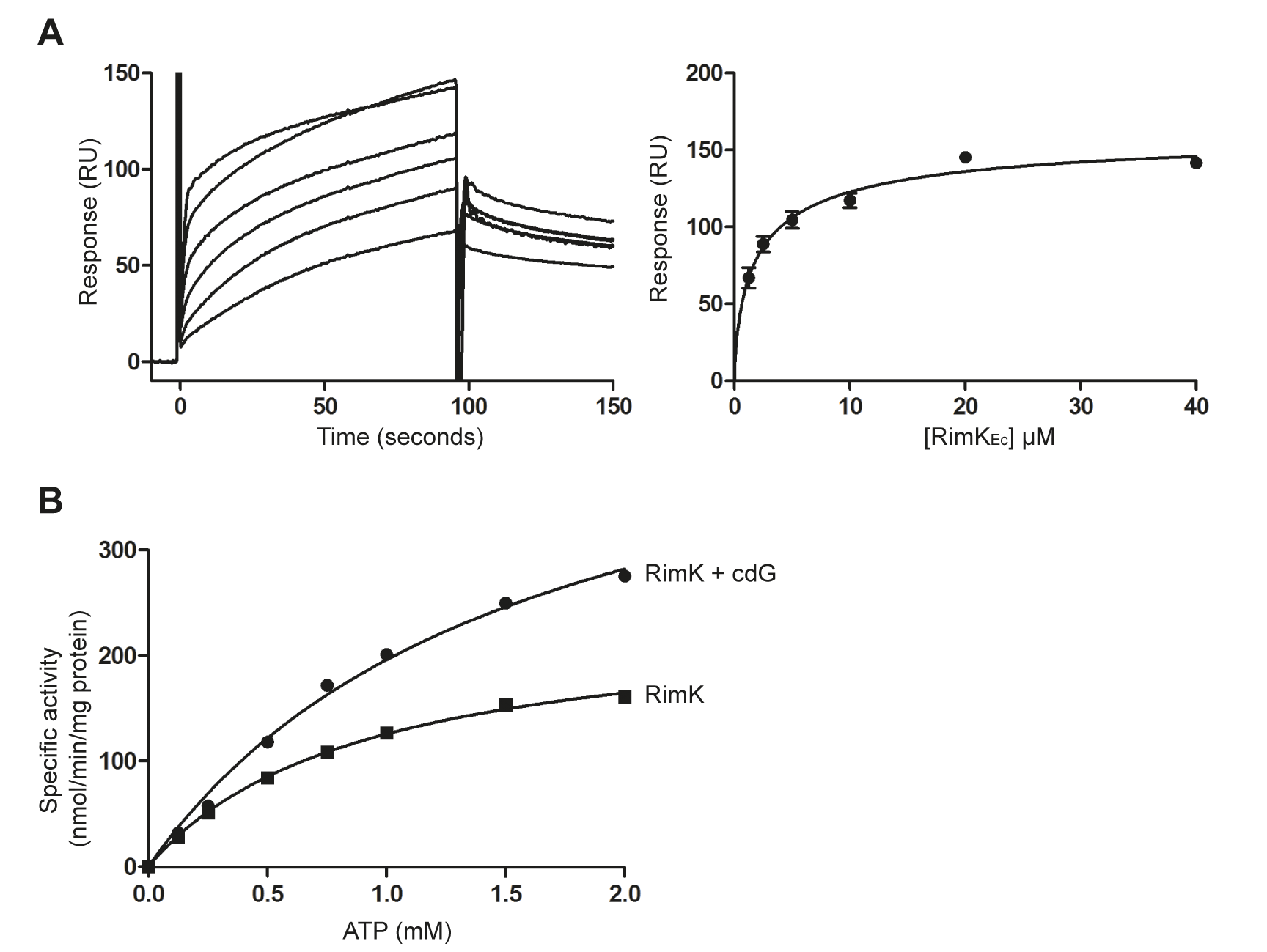

Supplement: S4 Fig — A. SPR sensorgram and affinity data for RimKEc binding to biotinylated cdG. A range of RimKEc concentrations was used (1.25, 2.5, 5, 10, 20, and 40 μM) and concentration replicates included as appropriate together with buffer only controls. Protein binding and dissociation phases are shown. For the affinity fit, binding responses were measured 4s before the end of the injection and Kd values for each protein calculated using BiaEvaluation software, and confirmed using GraphPad. B. ATPase activity of RimKPf incubated with glutamate and cdG. RimKPf specific activity (Vmax = 234.9 nmol ATP/min/mg protein) is shown for increasing concentrations of ATP (squares). Addition of 25 μM cdG (circles) increases Vmax to 471.0 nmol ATP/min/mg. (TIF) [file pgen.1005837.s004.tif]

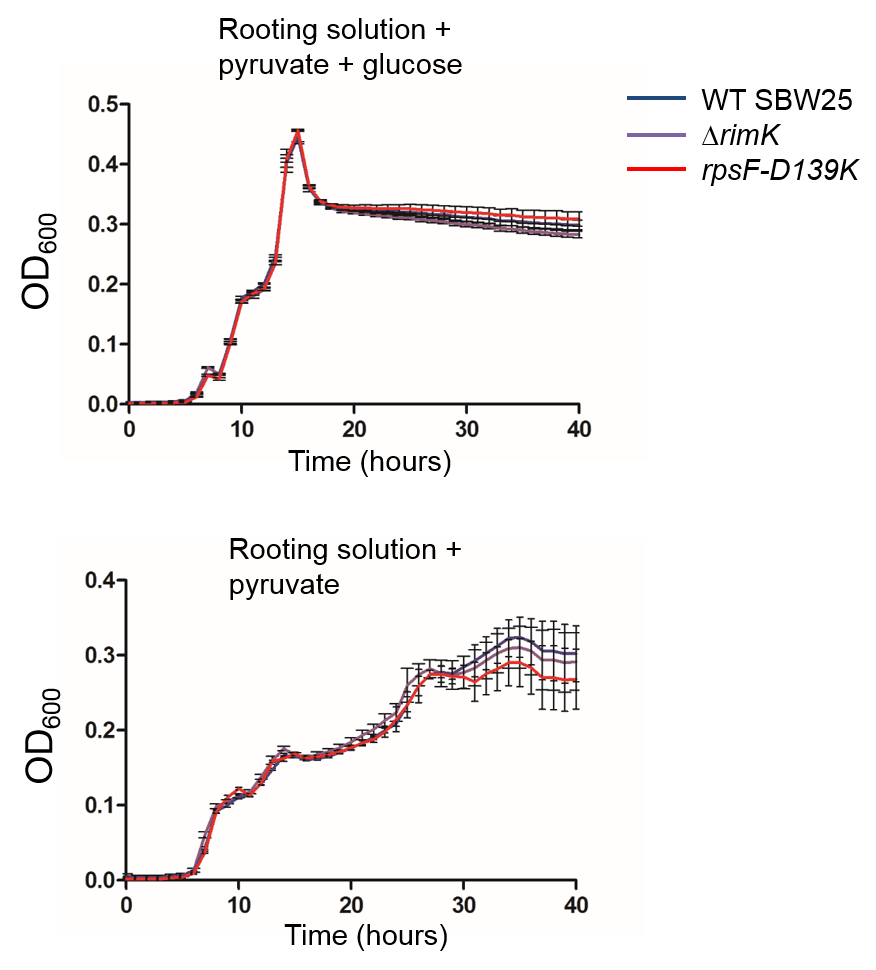

Supplement: S5 Fig — Growth curves for SBW25 WT, ΔrimK and rpsF-D139K in rooting solution + 0.4% pyruvate, ± 0.4% glucose. No significant differences in growth rate were seen between WT and rpsF-D139K in either condition. (TIF) [file pgen.1005837.s005.tif]
