# Supplementary material for: Adaptive Remodeling of the Bacterial Proteome by Specific Ribosomal Modification Regulates Pseudomonas Infection and Niche Colonisation
Source: PLoS Genet. 2016 Feb 4;12(2):e1005837. doi: 10.1371/journal.pgen.1005837 (PMC4741518; doi:10.1371/journal.pgen.1005837)
Supplement: S1 Table — (DOCX) [file pgen.1005837.s006.docx]

**S1 Table. Ribosomal proteins detected in SBW25 WT and ∆*rimK***

| Ribosomal Protein | Unique peptides | LFQ Intensity WT | LFQ Intensity ∆*rimK* | Ratio ∆*rimK*/WT |
| --- | --- | --- | --- | --- |
| L6 | 2 | 18239000 | 0 | N/A |
| L25 | 2 | 33561000 | 11365000 | 0.3386 |
| L9 | 2 | 28060000 | 10322000 | 0.3679 |
| S2 | 3 | 29145000 | 12901000 | 0.4426 |
| L18 | 4 | 28214000 | 13868000 | 0.4915 |
| L2 | 3 | 30056000 | 16083000 | 0.5351 |
| L7/L12 | 5 | 191000000 | 109940000 | 0.5756 |
| L13 | 4 | 44498000 | 25905000 | 0.5822 |
| L11 | 3 | 25486000 | 16406000 | 0.6437 |
| L1 | 2 | 30825000 | 20089000 | 0.6517 |
| L3 | 6 | 49709000 | 32435000 | 0.6525 |
| S5 | 2 | 25296000 | 16872000 | 0.6670 |
| S1 | 9 | 18831000 | 13476000 | 0.7156 |
| S10 | 2 | 25985000 | 20095000 | 0.7733 |
| L32 | 3 | 17074000 | 16308000 | 0.9551 |
