# Supplementary material for: Adaptive Remodeling of the Bacterial Proteome by Specific Ribosomal Modification Regulates Pseudomonas Infection and Niche Colonisation
Source: PLoS Genet. 2016 Feb 4;12(2):e1005837. doi: 10.1371/journal.pgen.1005837 (PMC4741518; doi:10.1371/journal.pgen.1005837)
Supplement: S2 Table — (DOCX) [file pgen.1005837.s007.docx]

**S2 Table. Up and Down-regulated Proteins in SBW25 ∆*rimK***

| **Downregulated in ∆*rimK*** | **WT/∆*rimK* Assay 1** | **WT/∆*rimK* Assay 2** | **Average** |
| --- | --- | --- | --- |
| Putative aldolase PFLU_4292 | **12.835** | **14.241** | 13.538 |
| Protein Hfq | **7.536** | **2.921** | 5.229 |
| Putative D-hydantoinase PFLU_3942 | **3.914** | **5.394** | 4.654 |
| 50S ribosomal protein L34 rpmH | **6.361** | **2.056** | 4.209 |
| 50S ribosomal protein L24 rplX | **5.283** | **2.701** | 3.992 |
| Putative uncharacterized protein PFLU_1522 | **4.997** | **2.567** | 3.782 |
| Putative uncharacterized protein PFLU_5262 | **5.363** | **1.878** | 3.621 |
| Putative uncharacterized protein PFLU_2951 | **3.464** | **3.436** | 3.450 |
| Putative outer membrane protein PFLU_1327 | **3.816** | **2.632** | 3.224 |
| Putative uncharacterized protein PFLU_3617 | **3.747** | **2.257** | 3.002 |
| 50S ribosomal protein L16 rplP | **3.570** | **1.965** | 2.768 |
| Putative regulatory protein PFLU_2993 | **3.401** | **1.928** | 2.665 |
| 30S ribosomal protein S15 rpsO | **3.650** | **1.635** | 2.643 |
| Phosphate starvation-inducible protein psiF | **2.085** | **3.159** | 2.622 |
| Transcriptional regulatory protein algP2 | **2.906** | **2.130** | 2.518 |
| Regulator of secondary metabolism RsmE PFLU_4165 | **3.841** | **1.181** | 2.511 |
| Transcriptional regulatory protein AlgP | **2.806** | **2.215** | 2.511 |
| DNA-binding protein HU1 hupA | **3.260** | **1.639** | 2.450 |
| Ubiquinol-cytochrome c reductase iron-sulfur subunit petA | **2.954** | **1.945** | 2.450 |
| 30S ribosomal protein S13 rpsM | **2.919** | **1.977** | 2.448 |
| Putative uncharacterized protein PFLU_5073 | **3.016** | **1.826** | 2.421 |
| 30S ribosomal protein S21 rpsU | **3.219** | **1.536** | 2.378 |
| Catalase katE | **1.634** | **3.110** | 2.372 |
| 2-nonaprenyl-3-methyl-6-methoxy-1,4-benzoquinol hydroxylase coq7 | **2.403** | **2.314** | 2.359 |
| Putative uncharacterized protein PFLU_0413 | **3.434** | **1.269** | 2.352 |
| 50S ribosomal protein L22 rplV | **2.778** | **1.863** | 2.321 |
| 50S ribosomal protein L6 rplF | **2.877** | **1.724** | 2.301 |
| NADH dehydrogenase I chain E PFLU_3821 | **2.469** | **2.093** | 2.281 |
| Putative uncharacterized protein PFLU_1746 | **2.662** | **1.878** | 2.270 |
| Integration host factor subunit beta ihfB | **2.604** | **1.933** | 2.269 |
| 30S ribosomal protein S18 rpsR | **2.802** | **1.704** | 2.253 |
| 30S ribosomal protein S9 rpsI | **2.494** | **2.006** | 2.250 |
| Putative uncharacterized protein PFLU_0038 | **2.489** | **1.965** | 2.227 |
| 30S ribosomal protein S19 rpsS | **2.827** | **1.542** | 2.185 |
| ATP-dependent protease subunit HslV | **2.373** | **1.946** | 2.160 |
| Putative uncharacterized protein PFLU_2493 | **2.727** | **1.581** | 2.154 |
| 50S ribosomal protein L18 rplR | **2.406** | **1.897** | 2.152 |
| 30S ribosomal protein S7 rpsG | **2.802** | **1.481** | 2.142 |
| 30S ribosomal protein S10 rpsJ | **2.648** | **1.555** | 2.102 |
| UPF0434 protein PFLU_3771 | **2.929** | **1.264** | 2.097 |
| 50S ribosomal protein L3 rplC | **2.450** | **1.714** | 2.082 |
| Osmotically inducible protein Y PFLU_5248 | **2.251** | **1.895** | 2.073 |
| DNA-binding protein HU-beta PFLU_3926 | **2.729** | **1.414** | 2.072 |
| Putative uncharacterized protein PFLU_5954 | **2.515** | **1.591** | 2.053 |
| Putative uncharacterized protein PFLU_4313 | **2.542** | **1.563** | 2.053 |
| 50S ribosomal protein L21 rplU | **2.194** | **1.876** | 2.035 |
| Putative short chain dehydrogenase/reductase PFLU_0654 | **1.871** | **2.145** | 2.008 |
|  | | | |
| **Upregulated in ∆*rimK*** | **∆*rimK*/WT Assay 1** | **∆*rimK*/WT Assay 2** | **Average** |
| UPF0234 protein PFLU_4927 | **2.041** | **1.992** | 2.016 |
| Putative uncharacterized protein PFLU_4689 | **1.838** | **2.222** | 2.030 |
| Putative uncharacterized protein PFLU_1037 | **1.905** | **2.174** | 2.039 |
| Pyrroline-5-carboxylate reductase PFLU_5764 | **2.045** | **2.079** | 2.062 |
| Putative secreted amidase PFLU_3453 | **2.169** | **1.957** | 2.063 |
| UPF0234 protein PFLU_4927 | **1.946** | **2.183** | 2.064 |
| Enolase 1 eno1 | **1.786** | **2.387** | 2.086 |
| Putative acyl carrier protein PFLU_3200 | **2.421** | **1.779** | 2.100 |
| Site-determining protein MinD PFLU_4370 | **1.880** | **2.358** | 2.119 |
| Phosphoserine aminotransferase serC | **2.247** | **1.992** | 2.120 |
| Putative NADP-dependent malic enzyme PFLU_0405 | **2.037** | **2.222** | 2.129 |
| Putative uncharacterized protein PFLU_5768 | **2.381** | **1.905** | 2.143 |
| Putative uncharacterized protein PFLU_4803 | **2.257** | **2.049** | 2.153 |
| Putative uncharacterized protein PFLU_1551 | **1.709** | **2.604** | 2.157 |
| 3-phosphoshikimate 1-carboxyvinyltransferase 2 aroA2 | **1.661** | **2.681** | 2.171 |
| Putative dehydrogenase PFLU_0670 | **2.625** | **1.718** | 2.171 |
| Aspartate aminotransferase PFLU_4460 | **2.075** | **2.283** | 2.179 |
| UTP--glucose-1-phosphate uridylyltransferase PFLU_2985 | **1.949** | **2.410** | 2.179 |
| Glutathione reductase PFLU_2986 | **2.232** | **2.146** | 2.189 |
| Succinylglutamate desuccinylase astE | **2.825** | **1.558** | 2.191 |
| Putative pterin-4-alpha-carbinolamine dehydratase PFLU_4459 | **2.433** | **1.961** | 2.197 |
| Nicotinate-nucleotide pyrophosphorylase nadC | **2.445** | **1.965** | 2.205 |
| Transcription elongation factor GreA 2 greA2 | **2.037** | **2.392** | 2.215 |
| Putative hydrolase PFLU_5827 | **1.931** | **2.506** | 2.218 |
| Putative uncharacterized protein PFLU_1158 | **1.965** | **2.481** | 2.223 |
| Putative uncharacterized protein PFLU_6006 | **2.817** | **1.637** | 2.227 |
| 3-deoxy-D-manno-octulosonate 8-phosphate phosphatase kdsC | **1.770** | **2.688** | 2.229 |
| Putative OmpA family lipoprotein PFLU_4907 | **2.874** | **1.587** | 2.230 |
| UDP-N-acetylmuramoyl-tripeptide--D-alanyl-D-alanine ligase murF | **2.227** | **2.257** | 2.242 |
| Putative ABC transport system, substrate-binding protein PFLU_2041 | **2.119** | **2.387** | 2.253 |
| Putative regulatory protein PFLU_1195 | **2.188** | **2.347** | 2.268 |
| Acyl carrier protein acpP | **2.183** | **2.364** | 2.274 |
| Dihydroorotase pyrC | **2.545** | **2.004** | 2.274 |
| Putative uncharacterized protein PFLU_2070 | **2.825** | **1.748** | 2.287 |
| Chaperone protein HscA homolog hscA | **1.972** | **2.604** | 2.288 |
| Putative uncharacterized protein PFLU_2888 | **2.304** | **2.273** | 2.288 |
| Aminomethyltransferase gcvT | **2.392** | **2.208** | 2.300 |
| Glucokinase PFLU_4963 | **2.950** | **1.653** | 2.301 |
| Aminomethyltransferase PFLU_4899 | **2.475** | **2.128** | 2.301 |
| Cold shock protein capB | **1.745** | **2.865** | 2.305 |
| Glutamate-1-semialdehyde 2,1-aminomutase hemL | **2.710** | **1.912** | 2.311 |
| Major cold shock protein cspA3 | **1.658** | **2.976** | 2.317 |
| Putative uncharacterized protein PFLU_1909 | **3.058** | **1.587** | 2.323 |
| Putative uncharacterized protein PFLU_5173 | **2.169** | **2.500** | 2.335 |
| Phosphoglucosamine mutase glmM | **2.985** | **1.689** | 2.337 |
| Putative uncharacterized protein PFLU_4879 | **2.404** | **2.342** | 2.373 |
| Probable transcriptional regulatory protein PFLU_3909 | **1.770** | **2.976** | 2.373 |
| Electron transfer flavoprotein alpha-subunit PFLU_4676 | **2.198** | **2.551** | 2.374 |
| Putative oxidoreductase PFLU_4576 | **2.841** | **1.946** | 2.393 |
| Putative GTP-binding protein PFLU_0729 | **2.519** | **2.278** | 2.398 |
| DNA-binding response regulator ompR | **2.183** | **2.625** | 2.404 |
| Haem utilisation protein PFLU_4969 | **2.041** | **2.770** | 2.405 |
| Putative uncharacterized protein PFLU_5550 | **2.833** | **1.984** | 2.408 |
| 3-oxoacyl-[acyl-carrier-protein] synthase 2 PFLU_3199 | **3.401** | **1.425** | 2.413 |
| Putative dehydrogenase PFLU_5606 | **3.367** | **1.481** | 2.424 |
| Trans-aconitate 2-methyltransferase tam | **2.237** | **2.618** | 2.427 |
| Putative ABC transport system, exported protein PFLU_0891 | **2.604** | **2.268** | 2.436 |
| Electron transfer flavoprotein beta-subunit PFLU_4677 | **2.342** | **2.564** | 2.453 |
| Biosynthetic arginine decarboxylase speA | **3.509** | **1.401** | 2.455 |
| Glutamate--tRNA ligase gltX | **3.356** | **1.563** | 2.459 |
| Peptide methionine sulfoxide reductase MsrA 3 msrA3 | **2.625** | **2.370** | 2.497 |
| Putative uncharacterized protein PFLU_4377 | **2.049** | **2.950** | 2.500 |
| Putative 6-phosphogluconolactonase PFLU_4837 | **2.632** | **2.375** | 2.503 |
| Putative magnesium chelatase protein PFLU_2671 | **2.404** | **2.618** | 2.511 |
| Putative uncharacterized protein PFLU_0903 | **2.564** | **2.463** | 2.514 |
| L-arabinose-binding periplasmic protein PFLU_4684 | **2.096** | **2.941** | 2.519 |
| Putative reductase PFLU_1621 | **1.984** | **3.067** | 2.526 |
| Phosphoribosylaminoimidazole-succinocarboxamide synthase purC | **2.564** | **2.488** | 2.526 |
| GTP cyclohydrolase-2 ribA | **2.288** | **2.770** | 2.529 |
| Putative sulfurylase PFLU_4624 | **2.564** | **2.500** | 2.532 |
| Putative exported heme receptor protein PFLU_4968 | **3.509** | **1.563** | 2.536 |
| Conserved hypothetical membrane protein PFLU_1216 | **1.808** | **3.268** | 2.538 |
| Malate synthase G glcB | **2.755** | **2.326** | 2.540 |
| Putative uncharacterized protein PFLU_3177 | **2.146** | **2.941** | 2.544 |
| Putative exported protein PFLU_3517 | **3.185** | **1.919** | 2.552 |
| Putative phage-related protein PFLU_0522 | **3.534** | **1.616** | 2.575 |
| Dihydrodipicolinate synthase 1 dapA | **2.336** | **2.825** | 2.581 |
| Isoleucine--tRNA ligase ileS | **3.802** | **1.387** | 2.595 |
| Putative hemin transport system, substrate-binding protein PFLU_5229 | **3.003** | **2.198** | 2.600 |
| Putative uncharacterized protein PFLU_1582 | **2.033** | **3.185** | 2.609 |
| Site-determining protein PFLU_4418 | **3.279** | **1.969** | 2.624 |
| Putative dehydrogenase PFLU_0663 | **2.257** | **3.003** | 2.630 |
| Protein TolB | **3.559** | **1.709** | 2.634 |
| Peptide methionine sulfoxide reductase MsrA 1 msrA | **2.445** | **2.825** | 2.635 |
| 3-isopropylmalate dehydrogenase leuB | **3.077** | **2.208** | 2.642 |
| Lactoylglutathione lyase PFLU_2991 | **2.353** | **2.941** | 2.647 |
| Type IV pilus response regulator/twitching mobility protein pilH | **1.773** | **3.521** | 2.647 |
| Putative isoprenoid biosynthesis-related protein elbB | **2.410** | **2.890** | 2.650 |
| Ferripyoverdine receptor fpvA | **3.953** | **1.359** | 2.656 |
| Putative uncharacterized protein PFLU_6020 | **3.125** | **2.203** | 2.664 |
| Orotate phosphoribosyltransferase pyrE | **3.367** | **1.976** | 2.672 |
| Tail-specific protease PFLU_2032 | **1.912** | **3.436** | 2.674 |
| Putative hydratase/isomerase protein PFLU_1440 | **3.584** | **1.818** | 2.701 |
| Ribosome-recycling factor frr | **2.232** | **3.185** | 2.708 |
| Transaldolase 1 talB | **2.427** | **3.003** | 2.715 |
| Putative uncharacterized protein PFLU_2551 | **3.846** | **1.610** | 2.728 |
| 2,3-bisphosphoglycerate-independent phosphoglycerate mutase gpmI | **2.611** | **2.865** | 2.738 |
| Phosphoenolpyruvate carboxykinase [ATP] pckA | **3.311** | **2.198** | 2.755 |
| Imidazole glycerol phosphate synthase subunit HisH | **2.494** | **3.021** | 2.757 |
| UDP-N-acetylglucosamine 1-carboxyvinyltransferase murA | **3.268** | **2.262** | 2.765 |
| Putative uncharacterized protein PFLU_5573 | **2.141** | **3.390** | 2.766 |
| UPF0345 protein PFLU_4399 | **2.336** | **3.226** | 2.781 |
| Putative uncharacterized protein PFLU_5479 | **2.033** | **3.546** | 2.789 |
| Phosphoribosylamine--glycine ligase purD | **3.322** | **2.268** | 2.795 |
| Phosphoglycerate kinase pgk | **2.415** | **3.185** | 2.800 |
| Alcohol dehydrogenase class III adhC | **2.857** | **2.770** | 2.814 |
| Putative uncharacterized protein PFLU_2570 | **2.488** | **3.145** | 2.816 |
| Putative uncharacterized protein PFLU_4598 | **2.075** | **3.584** | 2.829 |
| Acetyl-coenzyme A synthetase 1 acsA1 | **3.906** | **1.764** | 2.835 |
| 3-hydroxydecanoyl-[acyl-carrier-protein] dehydratase fabA | **3.195** | **2.475** | 2.835 |
| Branched-chain-amino-acid aminotransferase PFLU_3968 | **2.740** | **2.985** | 2.862 |
| Octaprenyl-diphosphate synthase PFLU_5172 | **3.106** | **2.625** | 2.865 |
| UDP-3-O-[3-hydroxymyristoyl] N-acetylglucosamine deacetylase lpxC | **2.604** | **3.175** | 2.889 |
| Putative uncharacterized protein PFLU_4149 | **3.745** | **2.058** | 2.901 |
| Probable thiol peroxidase tpx | **2.358** | **3.448** | 2.903 |
| Putative nitroreductase PFLU_1519 | **3.236** | **2.577** | 2.907 |
| Indole-3-glycerol phosphate synthase trpC | **2.433** | **3.413** | 2.923 |
| Pyridoxine/pyridoxamine 5~-phosphate oxidase pdxH | **3.205** | **2.660** | 2.932 |
| Putative uncharacterized protein PFLU_5950 | **2.387** | **3.484** | 2.935 |
| Glycine cleavage system H protein 2 gcvH | **2.183** | **3.731** | 2.957 |
| Glycine dehydrogenase [decarboxylating] gcvP | **4.630** | **1.294** | 2.962 |
| Putative alanyl-tRNA synthetase PFLU_4627 | **2.740** | **3.226** | 2.983 |
| Putative uncharacterized protein PFLU_3219 | **3.086** | **2.924** | 3.005 |
| Adenylate kinase adk | **2.740** | **3.279** | 3.009 |
| DNA gyrase subunit A gyrA | **4.673** | **1.351** | 3.012 |
| Putative hydrolase PFLU_1831 | **2.890** | **3.175** | 3.032 |
| Putative cobalamin biosynthesis-related protein PFLU_2669 | **3.096** | **3.003** | 3.049 |
| Putative glutathione S-transferase PFLU_2378 | **4.049** | **2.053** | 3.051 |
| Putative ABC transport system, exported protein PFLU_0376 | **2.976** | **3.135** | 3.055 |
| Putative uncharacterized protein PFLU_1231 | **4.717** | **1.462** | 3.089 |
| Biopolymer transport protein exbD | **4.444** | **1.736** | 3.090 |
| Bifunctional polymyxin resistance protein ArnA | **4.630** | **1.560** | 3.095 |
| Protein disulfide isomerase II PFLU_5007 | **3.509** | **2.688** | 3.098 |
| Peptidyl-prolyl cis-trans isomerase PFLU_3873 | **2.247** | **4.032** | 3.140 |
| Putative phosphomannomutase/phosphoglucomutase PFLU_5986 | **3.040** | **3.257** | 3.148 |
| Putative uncharacterized protein PFLU_2865 | **2.703** | **3.597** | 3.150 |
| Putative sulfurtransferase PFLU_5786 | **3.279** | **3.030** | 3.154 |
| GMP synthase [glutamine-hydrolyzing] guaA | **5.025** | **1.285** | 3.155 |
| Elongation factor P efp | **2.809** | **3.509** | 3.159 |
| Putative TonB-dependent receptor PFLU_1087 | **2.155** | **4.202** | 3.178 |
| Azurin azu | **3.937** | **2.481** | 3.209 |
| Putative ABC transport system, substrate-binding protein PFLU_2039 | **3.717** | **2.710** | 3.214 |
| Putative HIT domain-containing protein PFLU_5553 | **2.841** | **3.597** | 3.219 |
| Superoxide dismutase sodA | **2.857** | **3.584** | 3.221 |
| Transaldolase 2 tal2 | **3.521** | **2.967** | 3.244 |
| Putative uncharacterized protein PFLU_1890 | **4.329** | **2.160** | 3.244 |
| Thioredoxin trxA | **2.564** | **4.032** | 3.298 |
| Putative NADP-dependent glyceraldehyde-3-phosphate dehydrogenase PFLU_3844 | **5.208** | **1.401** | 3.304 |
| Phosphotransferase enzyme IIA component ptsN | **2.801** | **3.817** | 3.309 |
| Putrescine ABC transport system, substrate-binding periplasmic protein potF2 | **3.846** | **2.786** | 3.316 |
| Putative aldose 1-epimerase PFLU_2375 | **3.311** | **3.322** | 3.317 |
| Triosephosphate isomerase tpiA | **3.937** | **2.703** | 3.320 |
| Putative uncharacterized protein PFLU_4669 | **4.032** | **2.717** | 3.375 |
| Chaperone SurA | **3.650** | **3.106** | 3.378 |
| Putative sugar ABC transport system, lipoprotein PFLU_3117 | **3.247** | **3.534** | 3.390 |
| 1-(5-phosphoribosyl)-5-[(5-phosphoribosylamino)methylideneamino] imidazole-4-carboxamide isomerase hisA | **3.597** | **3.236** | 3.417 |
| Putative uncharacterized protein PFLU_5224 | **5.025** | **1.818** | 3.422 |
| Phage protein PFLU_2832 | **2.519** | **4.348** | 3.433 |
| Peptidyl-prolyl cis-trans isomerase PFLU_2635 | **2.326** | **4.566** | 3.446 |
| Chemotaxis response regulator CheY | **3.497** | **3.436** | 3.466 |
| Putative diaminobutyrate--2-oxoglutarate aminotransferase PFLU_3217 | **5.208** | **1.748** | 3.478 |
| Putative oxidoreductase PFLU_5859 | **3.390** | **3.571** | 3.481 |
| Putative ribose transport system, substrate-binding protein PFLU_4160 | **3.448** | **3.559** | 3.503 |
| 2-methylcitrate dehydratase PFLU_4628 | **5.780** | **1.258** | 3.519 |
| Putative uncharacterized protein PFLU_5830 | **4.310** | **2.778** | 3.544 |
| Phosphorylase glgP | **5.051** | **2.049** | 3.550 |
| Putative sugar-binding exported protein PFLU_3996 | **3.861** | **3.311** | 3.586 |
| Putative exported protein PFLU_1896 | **3.953** | **3.257** | 3.605 |
| Phosphoenolpyruvate carboxylase ppc | **5.988** | **1.299** | 3.643 |
| Putative peptidase PFLU_3649 | **4.785** | **2.545** | 3.665 |
| Threonine synthase thrC | **5.848** | **1.490** | 3.669 |
| Thiol:disulfide interchange protein DsbA | **2.941** | **4.425** | 3.683 |
| NAD dependent epimerase/dehydratase fnl2 | **5.848** | **1.567** | 3.708 |
| Putative hydrolase PFLU_5821 | **4.367** | **3.135** | 3.751 |
| Putative uncharacterized protein PFLU_5434 | **2.994** | **4.695** | 3.844 |
| Putative O-acetylserine (Thiol)-lyase PFLU_4625 | **4.608** | **3.155** | 3.881 |
| Putative iron utilisation protein PFLU_1089 | **4.255** | **3.509** | 3.882 |
| NAD-glutamate dehydrogenase PFLU_3504 | **5.988** | **1.825** | 3.906 |
| Putative amidase PFLU_3353 | **4.505** | **3.356** | 3.930 |
| Dipeptide ABC transport system, substrate-binding protein dppA1 | **4.717** | **3.195** | 3.956 |
| ATP synthase subunit b atpF | **6.329** | **1.590** | 3.959 |
| Putative acyl CoA oxidase PFLU_3198 | **6.329** | **1.592** | 3.961 |
| Putative uncharacterized protein PFLU_5582 | **6.410** | **1.761** | 4.085 |
| Putative glycine betaine/L-proline ABC transport system, substrate-binding protein PFLU_5680 | **4.032** | **4.184** | 4.108 |
| Putative exported protein PFLU_2914 | **3.968** | **4.292** | 4.130 |
| Putative phage-related protein PFLU_0523 | **6.849** | **1.587** | 4.218 |
| Conserved hypothetical exported protein PFLU_0091 | **4.484** | **3.984** | 4.234 |
| Putative non-ribosomal peptide synthetase PFLU_3224 | **5.682** | **2.793** | 4.238 |
| Putative ABC transport system, substrate-binding protein PFLU_1213 | **5.051** | **3.448** | 4.249 |
| Putative exported protein PFLU_3741 | **5.000** | **3.610** | 4.305 |
| Putative oxidoreductase PFLU_0041 | **5.952** | **2.747** | 4.350 |
| Putative non-ribosomal peptide synthetase PFLU_3222 | **6.711** | **2.155** | 4.433 |
| Maltose-binding protein PFLU_4846 | **5.682** | **3.472** | 4.577 |
| Putative L-ornithine 5-monooxygenase PFLU_3975 | **4.274** | **5.348** | 4.811 |
| Putative non-ribosomal peptide synthetase PFLU_3225 | **7.463** | **2.237** | 4.850 |
| Putative amino acid ABC transport system, substrate-binding protein PFLU_1000 | **5.495** | **4.292** | 4.893 |
| Putative membrane protein PFLU_4501 | **5.848** | **4.630** | 5.239 |
| Putative pyoverdine synthetase F PFLU_2547 | **5.650** | **4.831** | 5.240 |
| Putative hydrolase PFLU_1856 | **6.711** | **3.817** | 5.264 |
| Glutamate/aspartate ABC transport system, periplasmic binding protein gltI | **6.452** | **4.167** | 5.309 |
| Pyoverdin synthetase J PvdJ | **7.752** | **2.933** | 5.342 |
| Putative histidine-binding periplasmic protein PFLU_4765 | **5.650** | **5.348** | 5.499 |
| Putative ABC transport system, exported protein PFLU_0246 | **5.747** | **5.263** | 5.505 |
| Putative aminotransferase PFLU_5135 | **9.524** | **1.645** | 5.584 |
| Putative uncharacterized protein PFLU_3504 | **9.524** | **1.661** | 5.592 |
| Putative amino acid transport system, substrate-binding protein PFLU_1311 | **6.098** | **5.291** | 5.694 |
| Putative rhizopine-binding ABC transporter protein PFLU_2583 | **5.848** | **5.587** | 5.717 |
| Aromatic-amino-acid aminotransferase PFLU_4209 | **8.000** | **3.731** | 5.866 |
| Putative ubiquinol--cytochrome C reductase, cytochrome C1 PFLU_0843 | **9.259** | **3.003** | 6.131 |
| Biopolymer transport membrane protein exbB | **9.434** | **3.115** | 6.275 |
| Putative branched amino acid ABC transport system, substrate-binding protein livJ2 | **6.667** | **6.024** | 6.345 |
| Dipeptide ABC transport system, substrate-binding protein dppA2 | **8.264** | **4.444** | 6.354 |
| Putative amino acid ABC transport system, membrane protein PFLU_0313 | **6.803** | **6.211** | 6.507 |
| Putrescine ABC transport system, substrate-binding periplasmic protein potF1 | **6.667** | **6.452** | 6.559 |
| Putative exported protein PFLU_0215 | **5.405** | **7.874** | 6.640 |
| Glucose-6-phosphate isomerase pgi | **12.195** | **1.779** | 6.987 |
| Diaminobutyrate--2-oxoglutarate aminotransferase PFLU_4378 | **7.519** | **6.623** | 7.071 |
| Putative D-methionine ABC transport system, substrate-binding protein PFLU_0068 | **7.576** | **6.623** | 7.099 |
| Dipeptide ABC transport system, substrate-binding protein dppA3 | **9.346** | **5.525** | 7.435 |
| Branched amino acid ABC transport system, substrate-binding protein livJ1 | **8.850** | **7.092** | 7.971 |
| Putative membrane protein PFLU_5076 | **13.889** | **2.294** | 8.091 |
| Molybdate-binding periplasmic protein PFLU_2971 | **9.259** | **7.042** | 8.151 |
| Pyoverdine synthetase I pvdI | **12.821** | **3.534** | 8.177 |
| Aspartate-semialdehyde dehydrogenase asd | **11.765** | **4.902** | 8.333 |
| UPF0312 protein PFLU_5725 | **8.065** | **8.621** | 8.343 |
| Putative membrane protein PFLU_0832 | **14.706** | **4.348** | 9.527 |
| Putative ABC transport system, periplasmic protein PFLU_6091 | **8.696** | **19.231** | 13.963 |
