# Supplementary material for: Adaptive Remodeling of the Bacterial Proteome by Specific Ribosomal Modification Regulates Pseudomonas Infection and Niche Colonisation
Source: PLoS Genet. 2016 Feb 4;12(2):e1005837. doi: 10.1371/journal.pgen.1005837 (PMC4741518; doi:10.1371/journal.pgen.1005837)
Supplement: S3 Table — (DOCX) [file pgen.1005837.s008.docx]

**S3 Table. Up-regulated Proteins in SBW25 ∆*hfq***

| Up-regulated in ∆*hfq* | Assay 1 | Assay 2 | Average |
| --- | --- | --- | --- |
| Putative methyltransferase pflu_2308 | 146.94 | 136.2 | 143.09 |
| Putative hydroxyacid dehydrogenase pflu_2307 | 217.77 | 58.3 | 119.66 |
| Ferripyoverdine receptor fpva | 82.103 | 68.26 | 77.437 |
| Maltoporin pflu_5038 | 84.599 | 55.79 | 71.572 |
| Gtp-binding protein typa | 109.29 | 18.67 | 57.928 |
| Putative response regulator in two-component regulatory system with zras, regulates zrap expression (ebp family) pflu_5237 | 8.1689 | 74.61 | 51.74 |
| Putative aminotransferase pflu_5135 | 38.254 | 154.9 | 49.455 |
| Putative electron transport protein pflu_4678 | 37.046 | 28.75 | 33.47 |
| Putative outer membrane protein pflu_1450 | 37.711 | 22.94 | 31.4 |
| Putative oxidoreductase pflu_2628 | 28.354 | 12.61 | 20.916 |
| Superoxide dismutase soda | 28.141 | 1.477 | 11.963 |
| Putative dipeptidase pflu_2550 | 10.79 | 14.92 | 11.933 |
| Acetyl-coenzyme a synthetase 2 acsa2 | 19.801 | 3.443 | 10.974 |
| Pyruvate dehydrogenase e1 component acee1 | 11.106 | 6.065 | 10.435 |
| Putative gluconolactonase pflu_3458 | 13.531 | 4.435 | 10.043 |
| Putative iclr-family regulatory protein pflu_2305 | 14.551 | 4.856 | 9.843 |
| Aconitate hydratase 2 pflu_3489 | 10.847 | 5.958 | 9.6125 |
| Putative short-chain dehydrogenase/oxidoreductase pflu_3171 | 12.459 | 1.005 | 8.8325 |
| Atp synthase subunit delta atph | 793.08 | 0.134 | 8.5186 |
| 2-oxoglutarate dehydrogenase e1 component suca | 13.204 | 3.197 | 8.5126 |
| Putative lysr-family regulatory protein pflu_1118 transcriptional regulator metr | 11.634 | 3.858 | 7.8214 |
| Putative regulatory protein pflu_6073 | 10.924 | 4.944 | 7.6341 |
| Putative exported protein pflu_3741 carbohydrate abc transporter substrate-binding protein, cut1 family | 10.014 | 5.207 | 7.5975 |
| Fumarate hydratase class ii 1 fumc1 | 9.1342 | 5.424 | 7.474 |
| Nadh dehydrogenase ii ndh | 9.4107 | 6.081 | 7.4164 |
| Putative arac family regulatory protein pflu_4760 | 11.131 | 2.318 | 7.0896 |
| Isocitrate dehydrogenase [nadp] pflu_3809 | 7.0663 | 6.287 | 6.8346 |
| Sigma factor negative regulatory protein muca | 25.642 | 0.067 | 6.787 |
| Aconitate hydratase 1 acna | 8.8573 | 1.431 | 6.7814 |
| Trna-specific 2-thiouridylase mnma | 19.065 | 3.06 | 6.7146 |
| Cold-shock dead-box protein a dead | 37.724 | 4.679 | 6.5039 |
| Putative abc transport system, substrate-binding protein pflu_2041 | 8.9727 | 3.892 | 6.2691 |
| Proline--trna ligase pros | 9.7793 | 2.126 | 6.231 |
| Putative uncharacterized protein pflu_0923 | 8.6087 | 2.509 | 6.1777 |
| Phosphoenolpyruvate carboxykinase [atp] pcka | 5.9892 | 6.202 | 6.1707 |
| Putative uncharacterized protein pflu_4552 | 6.3653 | 5.695 | 6.0953 |
| Transcription termination factor pflu_5254 | 7.9868 | 4.113 | 5.9406 |
| Probable atp-binding abc transporter protein pflu_3746 | 8.8531 | 3.131 | 5.9143 |
| Probable septum site-determining protein minc | 12.217 | 1.597 | 5.9136 |
| Putative exported protein pflu_0688 | 5.1875 | 7.169 | 5.7758 |
| Putative d-hydantoinase pflu_3942 | 3.1583 | 8.647 | 5.7522 |
| L-serine dehydratase pflu_4898 | 4.8831 | 6.875 | 5.7181 |
| Putative atp-binding abc transporter protein pflu_3745 | 6.5736 | 3.645 | 5.6058 |
| Gntr-family transcriptional regulator pflu_3295 | 7.9913 | 2.87 | 5.4281 |
| Succinyl-diaminopimelate desuccinylase dape | 21.767 | 2.16 | 5.4235 |
| Putative uncharacterized protein pflu_6020 | 1.5101 | 7.937 | 5.3927 |
| Putative aldolase pflu_5151 | 10.174 | 1.499 | 5.3475 |
| Putative aldolase pflu_4292 | 11.043 | 1.481 | 5.3015 |
| Atp-dependent chaperone protein clpb | 8.1296 | 1.555 | 5.2498 |
| Glutamine synthetase glna | 2.6447 | 7.528 | 5.2288 |
| Ribosomal rna small subunit methyltransferase j rsmj | 6.7306 | 3.608 | 5.1775 |
| N-carbamyl-l-amino acid amidohydrolase pfl_2550 | 7.0352 | 3.53 | 5.1674 |
| D-xylose abc transport system, substrate-binding periplasmic protein xylf | 4.718 | 8.557 | 5.1445 |
