# Supplementary material for: Adaptive Remodeling of the Bacterial Proteome by Specific Ribosomal Modification Regulates Pseudomonas Infection and Niche Colonisation
Source: PLoS Genet. 2016 Feb 4;12(2):e1005837. doi: 10.1371/journal.pgen.1005837 (PMC4741518; doi:10.1371/journal.pgen.1005837)
Supplement: S4 Table — (DOCX) [file pgen.1005837.s009.docx]

**S4 Table. Strains and Plasmids**

| Strains | Description | Reference |
| --- | --- | --- |
| Pseudomonas |  |  |
| SBW25 | Environmental *P. fluorescens* isolate | [1] |
| SBW25 ∆rimA | SBW25 with *rimA* (*PFLU_0263*) deleted | This study |
| SBW25 ∆rimB | SBW25 with *rimB* (*PFLU_0262*) deleted | This study |
| SBW25 ∆rimK | SBW25 with *rimK* (*PFLU_0261*) deleted | This study |
| SBW25 ∆hfq | SBW25 with *hfq* (*PFLU_0520*) deleted | This study |
| SBW25 rpsF-D139K | SBW25 with *rpsF* (PFLU_0533) D139K mutant allele | This study |
| ∆rimA Tn7::rimA | SBW25 ∆*rimA* with *rimA* inserted at the att::Tn7 locus | This study |
| ∆rimB Tn7::rimB | SBW25 ∆*rimB* with *rimB* inserted at the att::Tn7 locus | This study |
| ∆rimK Tn7::rimK | SBW25 ∆*rimK* with *rimK* inserted at the att::Tn7 locus | This study |
| Pto DC3000 | Rif^R^ derivative of *P. syringae* pv. Tomato NCPPB 1106 | [2] |
| Pto DC3000 ∆rimK | DC3000 with *rimK* (*PSPTO_0234*) deleted | This study |
| PA01 | Wild-type *P. aeruginosa* | [3] |
| PA01 ∆rimK | PA01 with *rimK* (*PA5197*) deleted | This study |
| E. coli |  |  |
| BL21-(DE3) | Sm^R^, K12 *recF143 lacI^q^ lacZΔ.M15*, *xylA* | Novagen |
| DH5α | *endA*1, *hsdR*17(r_K_-m_K_+), *supE*44, *recA*1, *gyrA* (Nal^r^), *relA*1, Δ(*lacIZYA-argF*)U169, *deoR*, Φ80*dlacΔ(lacZ)M15* | [4] |
| Plasmids |  |  |
| pME6032 | Tet^R^, P_K_, 9.8 kb pVS1 derived shuttle vector | [5] |
| pME-rimA/B/K vectors | pME6032 with SBW25 *rimA/B/K* as *Eco*RI*-Kpn*I fragments | This study |
| pSUB11 | Amplification vector for *flag-*FRT-Kan^R^-FRT cassette | [6] |
| pFLP2 | Amp^R^, FRT cassette excision vector | [7] |
| pME3087 | Tet^R^, suicide vector; ColE1-replicon, IncP-1, Mob | [8] |
| pME3087- rim/hfq/rpsF vectors | pME3087 with *rim/hfq/rpsF* alleles as *Eco*RI*-Bam*HI fragments | This study |
| pUC18T-mini-Tn7T-Gm | Amp^R^, Gm^R^, Tn7 insertion vector | [9] |
| rimABK-Tn7 vectors | pUC18T-mini-Tn7T-Gm with rimABK genes as HindIII- BamHI fragments. | This study |
| pIJ-11-282 | pJP2 derivative with *luxCDABE* cassette expressed from *nptII* promoter. Luminescent marker plasmid | [10] |
| pETNdeM-11 | Km^R^, purification vector, N-terminal His_6_-tag | [11] |
| pETM11-rpsF | pET*Nde*M-11 with *rpsF* alleles as *Nde*I-*Xho*I fragments | This study |
| pET42b(+) | Km^R^, purification vector, C-terminal His_6_-tag | Novagen |
| pET42b(+)-rimA/B/K | pET42b(+) with *rimA/B/K* alleles as *Nde*I-*Xho*I fragments | This study |

1. Rainey PB, Bailey MJ. Physical and genetic map of the Pseudomonas fluorescens SBW25 chromosome. Mol Microbiol. 1996;19(3):521-33. Epub 1996/02/01. PubMed PMID: 8830243.

2. Cuppels DA. Generation and Characterization of Tn5 Insertion Mutations in Pseudomonas syringae pv. tomato. Appl Environ Microbiol. 1986;51(2):323-7. Epub 1986/02/01. PubMed PMID: 16346988; PubMed Central PMCID: PMC238867.

3. Holloway BW. Genetic recombination in Pseudomonas aeruginosa. J Gen Microbiol. 1955;13(3):572-81. PubMed PMID: 13278508.

4. Woodcock DM, Crowther PJ, Doherty J, Jefferson S, DeCruz E, Noyer-Weidner M, et al. Quantitative evaluation of Escherichia coli host strains for tolerance to cytosine methylation in plasmid and phage recombinants. Nucleic Acids Res. 1989;17(9):3469-78. PubMed PMID: 2657660.

5. Heeb S, Itoh Y, Nishijyo T, Schnider U, Keel C, Wade J, et al. Small, stable shuttle vectors based on the minimal pVS1 replicon for use in gram-negative, plant-associated bacteria. Molecular plant-microbe interactions : MPMI. 2000;13(2):232-7. PubMed PMID: 10659714.

6. Yu D, Ellis HM, Lee EC, Jenkins NA, Copeland NG, Court DL. An efficient recombination system for chromosome engineering in Escherichia coli. Proc Natl Acad Sci U S A. 2000;97(11):5978-83. PubMed PMID: 10811905.

7. Hoang TT, Karkhoff-Schweizer RR, Kutchma AJ, Schweizer HP. A broad-host-range Flp-FRT recombination system for site-specific excision of chromosomally-located DNA sequences: application for isolation of unmarked Pseudomonas aeruginosa mutants. Gene. 1998;212(1):77-86. PubMed PMID: 9661666.

8. Voisard C, Bull CT, Keel C, Laville J, Maurhofer M, U S. Biocontrol of root diseases by Pseudomonas fluorescens CHA0: current concepts and experimental approaches. O'Gara F, Dowling DN, Boesten B (eds) Molecular Ecology of Rhizosphere Microorganisms 1994:67-89.

9. Choi KH, Gaynor JB, White KG, Lopez C, Bosio CM, Karkhoff-Schweizer RR, et al. A Tn7-based broad-range bacterial cloning and expression system. Nature methods. 2005;2(6):443-8. PubMed PMID: 15908923.

10. Frederix M, Edwards A, Swiderska A, Stanger A, Karunakaran R, Williams A, et al. Mutation of praR in Rhizobium leguminosarum enhances root biofilms, improving nodulation competitiveness by increased expression of attachment proteins. Mol Microbiol. 2014;93(3):464-78. doi: 10.1111/mmi.12670. PubMed PMID: 24942546; PubMed Central PMCID: PMCPMC4149787.

11. Little R, Salinas P, Slavny P, Clarke TA, Dixon R. Substitutions in the redox-sensing PAS domain of the NifL regulatory protein define an inter-subunit pathway for redox signal transmission. Mol Microbiol. 2011;82(1):222-35. Epub 2011/08/23. doi: 10.1111/j.1365-2958.2011.07812.x. PubMed PMID: 21854469.
