# Supplementary material for: Adaptive Remodeling of the Bacterial Proteome by Specific Ribosomal Modification Regulates Pseudomonas Infection and Niche Colonisation
Source: PLoS Genet. 2016 Feb 4;12(2):e1005837. doi: 10.1371/journal.pgen.1005837 (PMC4741518; doi:10.1371/journal.pgen.1005837)
Supplement: S5 Table — (DOCX) [file pgen.1005837.s010.docx]

**S5 Table. Primers**

| Number/name | Sequence (5’→3’) | Description |
| --- | --- | --- |
| 1/ PFLU0261-OEFor | CGCCAATTGATGAAGATTGCTGTGCTG | SBW25 *rimK* overexpression, forward primer |
| 2/ PFLU0261-OERev | AAGGTACCTCAGCCCTTGCCTTTGGTC | SBW25 *rimK* overexpression, reverse primer |
| 3/ PFLU0262-OEFor | CGGAATTCTTGAAGACATTTGACC | SBW25 *rimB* overexpression, forward primer |
| 4/ PFLU0262-OERev | TTGGTACCTCATGCAGCACCTGGGGCAG | SBW25 *rimB* overexpression, reverse primer |
| 5/ PFLU0263-OEFor | CAACAATTGGTGACCGACTTCCCCCTTTC | SBW25 *rimA* overexpression, forward primer |
| 6/ PFLU0263-OERev | GAGGTACCCGGTCAATTCGCCCAGG | SBW25 *rimA* overexpression, reverse primer |
| 7/ 0261flagFor | AAGAATGGCGGGCCGAATATGACTCGGACCAAAGGCAAGGGCGACTACAAAGACCATGACGG | flag-tag sequence for SBW25 *rimK*, forward primer |
| 8/ 0262flagFor | GCCGTGTTCCCGGTCTCCACTATTTCTGCCCCAGGTGCTGCAGACTACAAAGACCATGACGG | flag-tag sequence for SBW25 *rimB*, forward primer |
| 9/ 0263flagFor | TTCAACGCCCTGGCCGAGGTTTCACCCCAGGCCTGGGCGAATGACTACAAAGACCATGACGG | flag-tag sequence for SBW25 *rimA*, forward primer |
| 10/ pME6032M2Rev | CTAGTCCGAGGCCTCGAGATCTATCGATGCATGCCATGGTACCCATATGAATATCCTCCTTAG | flag-tag sequence for SBW25 *rimK*, reverse primer |
| 11/ 0261NdeFor | CAGAAGCCATATGAAGATTGCTGTGCTGTCG | SBW25 *rimK* purification, forward primer |
| 12/ 0261XhoRev | GGTACTCGAGGCCCTTGCCTTTGGTCCGAGTC | SBW25 *rimK* purification, reverse primer |
| 13/ 0262NdeFor | CAGAAGCCATATGAAGACATTTGACCATTTG | SBW25 *rimB* purification, forward primer |
| 14/ 0262XhoRev2 | GGTACTCGAGTCATGCAGCACCTGGGGC | SBW25 *rimB* purification, reverse primer |
| 15/ 0263NdeFor | CAGAAGCCATATGACCGACTTCCCCCTTTCAC | SBW25 *rimA* purification, forward primer |
| 16/ 0263XhoRev2 | GGTACTCGAGTCAATTCGCCCAGGCCTGGGG | SBW25 *rimA* purification, reverse primer |
| 17/ PFLU0261UPF | CGGGATCCTATTGGTTCGCCAAGC | *SBW25 rimK* deletion; forward primer, upstream |
| 18/ PFLU0261UPR | CGTCTAGACAGACGCGGGTTTCGCGAC | SBW25 *rimK* deletion; reverse primer, upstream |
| 19/ PFLU0261DNF | CGTCTAGATGATTAAACCCTGCTTTTG | SBW25 *rimK* deletion; forward primer, downstream |
| 20/ PFLU0261DNR | CGGAATTCGACGGACTTGGTGCGTG | SBW25 *rimK* deletion; reverse primer, downstream |
| 21/ 0533NdeFor | CAGAAGCCATATGCGTCATTACGAAATC | SBW25 *rpsF* purification, forward primer |
| 22/ 0533XhoRev2 | GGTACTCGAGTTACTCGTCAGCGTTATCGC | SBW25 *rpsF* purification, reverse primer |
| 23/ PSPTO234UPF | CGGGATCCCATATCCCTGATGCAAG | DC3000 *rimK* deletion; forward primer, upstream |
| 24/ PSPTO234UPR | CGTCTAGACGAAAGCACAGCGATCTTC | DC3000 *rimK* deletion; reverse primer, upstream |
| 25/ PSPTO234DNF | CGTCTAGAATGACCCGCACCAAGG | DC3000 *rimK* deletion; forward primer, downstream |
| 26/ PSPTO234DNR | CGGAATTCATATTACTGATCCACC | DC3000 *rimK* deletion; reverse primer, downstream |
| 27/ ColiRimKNdeFor | CAGAAGCCATATGAAAATTGCCATATTG | *E coli* *rimK* purification, forward primer |
| 28/ ColiRimKXhoRev1 | GGTACTCGAGACCACCCGTTTTCAGGC | *E. coli rimK* purification, reverse primer |
| 29/ ColiRpsFNdeFor | CAGAAGCCATATGCGTCATTACGAAATCGTT | *E coli* *rpsF* purification, forward primer |
| 30/ ColiRpsXhoRev2 | GGTACTCGAGTTACTCTTCAGAATCCCCAGC | *E. coli rpsF* purification, reverse primer |
| 31/ 5592RT-PCR1 | CAACGAAGTAGACGAAAGCTG | SBW25 *rpoD* forward primer for cDNA synthesis |
| 32/ 5592RT-PCR2 | GACGGTTGATGTCCTTGATCTC | SBW25 *rpoD* reverse primer for cDNA synthesis |
| 33/ 0261RT-PCR1 | GCAAGCCGCTGGAAGGGTTTGATG | SBW25 *rimK* forward primer for cDNA synthesis |
| 34/ 0261RT-PCR2 | GCAACTGCAGCGAACGCAACTTG | SBW25 *rimK* reverse primer for cDNA synthesis |
| 35/ 4165RT-PCR1 | GTCCATGCTTATACTCAC | SBW25 *rsmE* forward primer for cDNA synthesis |
| 36/ 4165RT-PCR2 | GATGCGCTGGTAGATCTCC | SBW25 *rsmE* reverse primer for cDNA synthesis |
| 37/ 0520RT-PCR1 | GCTACAAGACCCTTACTTG | SBW25 *hfq* forward primer for cDNA synthesis |
| 38/ 0520RT-PCR2 | GTGCTTGTATACCATCTGGC | SBW25 *hfq* reverse primer for cDNA synthesis |
| 39/ 3926RT-PCR1 | GAACTGATTGATGCTATCGC | SBW25 HU-β forward primer for cDNA synthesis |
| 40/ 3926RT-PCR2 | GTCAGTCACAGAGAAAGTAC | SBW25 HU-β reverse primer for cDNA synthesis |
| 41/ PFLU0068RT-For | TCAAGGACATCACTGGCAAC | SBW25 PFLU_0068 forward primer for cDNA synthesis |
| 42/ PFLU0068RT-Rev | GTTCACGTAAGGCGAGTCGT | SBW25 PFLU_0068 reverse primer for cDNA synthesis |
| 43/ PFLU2543RT-For | GAAAACGACCTGCAGAAAGC | SBW25 *pvdL* forward primer for cDNA synthesis |
| 44/ PFLU2543RT-Rev | CACGCTCTGGTACTGGAACA | SBW25 *pvdL* reverse primer for cDNA synthesis |
| 45/ PFLU3222RT-For | CGAGTGGATCAATGTGCAAC | SBW25 PFLU_3222 forward primer for cDNA synthesis |
| 46/ PFLU3222RT-Rev | GGCGTGGAGTGGTAGATGTT | SBW25 PFLU_3222 reverse primer for cDNA synthesis |
| 47/ PFLU5505RT-For | ATTGCAGGCGTTAACATTCC | SBW25 *rpsM*  forward primer for cDNA synthesis |
| 48/ PFLU5505RT-Rev | CAACAGCTCAATCTGCTCGT | SBW25 *rpsM*  reverse primer for cDNA synthesis |
| 49/ PFLU5516RT-For | CCGGCAAAGACAAAGGTAAG | SBW25 *rplX*  forward primer for cDNA synthesis |
| 50/ PFLU5516RT-Rev | CCAGTGGAGCTTCTTTTTCG | SBW25 *rplX*  reverse primer for cDNA synthesis |
| 51/ PFLU6091RT-For | GGTTACCAGCTTCAGCATCC | SBW25 PFLU_6091 forward primer for cDNA synthesis |
| 52/ PFLU6091RT-Rev | ATGATGACCTTGGCCTTGAG | SBW25 PFLU_6091 reverse primer for cDNA synthesis |
| 53/ PFLU0262UPF | CGGGATCCGTGCTGGACCAGGTCAAC | SBW25 *rimB* deletion; forward primer, upstream |
| 54/ PFLU0262UPR | CGTCTAGACAAATGGTCAAATGTC | SBW25 *rimB* deletion; reverse primer, upstream |
| 55/ PFLU0262DNF | CGTCTAGAGCCCCAGGTGCTGCATG | SBW25 *rimB* deletion; forward primer, downstream |
| 56/ PFLU0262DNR | CGGAATTCATTGCCGCAATCACCTTG | SBW25 *rimB* deletion; reverse primer, downstream |
| 57/ PFLU0263UPF | CGGGATCCCTAGTCATCGTTGCCAC | SBW25 *rimA* deletion; forward primer, upstream |
| 58/ PFLU0263UPR | CGTCTAGACGAGGTGAGTGAAAGG | SBW25 *rimA* deletion; reverse primer, upstream |
| 59/ PFLU0263DNF | CGTCTAGATGACCGGTTACCCATATTG | SBW25 *rimA* deletion; forward primer, downstream |
| 60/ PFLU0263DNR | CGGAATTCCAACGTATCGATCACCAC | SBW25 *rimA* deletion; reverse primer, downstream |
| 61/ PA01rimKupXbaI | CGtctagaCGTTGCGAAGCGCCGATG | PA01 *rimK* deletion; forward primer, upstream |
| 62/ PA01rimKupXhoI | CCGctcgagCATtcagcacctgtggaatgg | PA01 *rimK* deletion; reverse primer, upstream |
| 63/ PA01rimKdnXhoI | ccgCTCGAGtgagccggaccagacaacg | PA01 *rimK* deletion; forward primer, downstream |
| 64/ PA01rimKdnKpnI | CGggtaccACCAGACCGCGGTGATGTC | PA01 *rimK* deletion; reverse primer, downstream |
| 65/ 0533D139KXhoRev | GGTACTCGAGTTACTCCTTAGCGTTATCGC | SBW25 *rimK-D139K* purification, forward primer |
| 66/ 0533XhoFor | CCGCTCGAGGATGCTGAACGTTGAGTGC | SBW25 *rpsF-D139K* mutation; forward primer, upstream |
| 67/ 0533EmutFor2 | CGATAACGCTAAGGAGTAATC | SBW25 *rpsF-D139K* mutation; forward mutagenic primer |
| 68/ 0533EmutRev2 | GATTACTCCTTAGCGTTATCG | SBW25 *rpsF-D139K* mutation; reverse mutagenic primer |
| 69/ 0533Reverse | GCGGATCCCAATCCCTTACTACTTGTC | SBW25 *rpsF-D139K* mutation; reverse primer, downstream |
| 70/ 16S-RT-PCR1 | CAGGCCTTGACATCCAATGA | SBW25 16S ribosomal RNA forward primer for cDNA synthesis |
| 71/ 16S-RT-PCR2 | TTAGAGTGCCCACCATGACG | SBW25 16S ribosomal RNA reverse primer for cDNA synthesis |
| 72/ rpsF-RT-PCR3 | CGATGCAGTGATCCGTAACC | SBW25 *rpsF* forward primer for cDNA synthesis |
| 73/ rpsF-RT-PCR4 | ATCGCTGTTGTCGCTGTCAT | SBW25 *rpsF* reverse primer for cDNA synthesis |
| 74/ TnRimup1Hind | GCACAAGCTTGATGCACCTTGCCACTCTC | SBW25 *rimA,B,K* complementation, forward primer |
| 75/ TnRimA1revBam | GGTAGGATCCTCAATTCGCCCAGGCCTGGGG | SBW25 *rimA* complementation, reverse primer |
| 76/ TnRimB1revBam | GGTAGGATCCTCATGCAGCACCTGGGGC | SBW25 *rimB* complementation, reverse primer |
| 77/ TnRimK1revBam | GGTAGGATCCTCAGCCCTTGCCTTTGGTCCG | SBW25 *rimK* complementation, reverse primer |
